# Mechanisms and technologies in cancer epigenetics

**DOI:** 10.3389/fonc.2024.1513654

**Published:** 2025-01-07

**Authors:** Zaki A. Sherif, Olorunseun O. Ogunwobi, Habtom W. Ressom

**Affiliations:** ^1^ Department of Biochemistry & Molecular Biology, Howard University College of Medicine, Washington, DC, United States; ^2^ Department of Biochemistry & Molecular Biology, Michigan State University, East Lansing, MI, United States; ^3^ Department of Oncology, Georgetown University Medical Center, Washington, DC, United States

**Keywords:** cancer, chromatin, DNA methylation, epigenetics, epigenetic therapy, histone modifications, non-coding RNAs, tumorigenesis

## Abstract

Cancer’s epigenetic landscape, a labyrinthine tapestry of molecular modifications, has long captivated researchers with its profound influence on gene expression and cellular fate. This review discusses the intricate mechanisms underlying cancer epigenetics, unraveling the complex interplay between DNA methylation, histone modifications, chromatin remodeling, and non-coding RNAs. We navigate through the tumultuous seas of epigenetic dysregulation, exploring how these processes conspire to silence tumor suppressors and unleash oncogenic potential. The narrative pivots to cutting-edge technologies, revolutionizing our ability to decode the epigenome. From the granular insights of single-cell epigenomics to the holistic view offered by multi-omics approaches, we examine how these tools are reshaping our understanding of tumor heterogeneity and evolution. The review also highlights emerging techniques, such as spatial epigenomics and long-read sequencing, which promise to unveil the hidden dimensions of epigenetic regulation. Finally, we probed the transformative potential of CRISPR-based epigenome editing and computational analysis to transmute raw data into biological insights. This study seeks to synthesize a comprehensive yet nuanced understanding of the contemporary landscape and future directions of cancer epigenetic research.

## 1 Introduction

Cancer, a complex and heterogeneous disease characterized by aberrant cellular proliferation and differentiation, continues to pose significant challenges to global health (1). Although genetic mutations and metabolic dysregulation play key roles in cancer, the last 10 years have seen a major shift in our understanding of cancer biology. Cancer epigenetics has emerged as a pivotal factor in tumor formation and progression (2, 3).

Epigenetics refers to heritable changes in gene expression that do not involve alterations in the underlying DNA sequence. The core epigenetic mechanisms are DNA methylation, histone modifications, chromatin remodeling, and regulation by non-coding RNAs. These molecular switches are critical for normal cellular processes such as development and differentiation (4). However, when they malfunction, they can sculpt the cancer landscape and play a substantial role in cancer initiation and progression.

Notably, aberrant DNA methylation was the first epigenetic abnormality identified in human cancers (5–7). Since this initial discovery, research has revealed that cancer cells frequently exhibit a significantly altered epigenetic profile compared to normal cells (8–10). The epigenome of cancer is characterized by extensive abnormalities, including global hypomethylation and promoter-specific hypermethylation. This epigenetic dysregulation can effectively silence tumor suppressor genes or amplify oncogenes, facilitating the development of hallmark characteristics of cancer, which are widely recognized in the field (11).

Recent advancements in cancer epigenetics have been significantly accelerated by novel technologies that enable analysis of the epigenome with unprecedented resolution (12). High-throughput sequencing, paired with sophisticated bioinformatics tools, currently allows researchers to map epigenetic modifications across entire genomes and connect these changes to cancer phenotypes. These technological advancements have enabled highly innovative research endeavors (13).

This review presents a comprehensive examination of the current state of cancer epigenetic research by emphasizing recent discoveries and emerging technologies. The major epigenetic mechanisms implicated in cancer-DNA methylation patterns, histone modifications, chromatin remodeling, and the roles of various non-coding RNAs will be explored. Of particular interest is the discussion of how cutting-edge technologies such as single-cell sequencing and multi-omics approaches are transforming our understanding of tumor heterogeneity and complex interactions within the tumor microenvironment (14, 15).

As we navigate the intricate landscape of cancer epigenetics, this review will not only summarize key findings but also identify gaps in our current knowledge and indicate future research directions. In this context, we will also explore the clinical applications of epigenetic research, such as the development of epigenetic biomarkers for the early detection and prognosis of cancer. Unlike genetic mutations, epigenetic changes are potentially reversible, rendering epigenetic therapy a promising approach against cancer (16–18).

## 2 Methods

This comprehensive review was conducted using a systematic literature search strategy to identify relevant studies on cancer epigenetics focusing on recent advances in mechanisms, biomarkers, and technologies.

### 2.1 Search strategy

We performed a comprehensive search of electronic databases including NCBI’s PubMed, Thomson Reuters’ Web of Science, Scopus, and Google Scholar. The search covered articles published from January 2018 to August 2024 using the following key terms and their combinations:

- “Cancer epigenetics”
- “DNA methylation” AND “cancer”
- “Histone modifications” AND “cancer”
- “Chromatin remodeling” AND “cancer”
- “Non-coding RNA” AND “cancer”
- “Epigenetic biomarkers”
- “Single-cell epigenomics”
- “Multi-omics” AND “cancer”
- “Spatial epigenomics”
- “CRISPR epigenome editing”

We also combined keywords, Boolean operators (AND, OR, and NOT), and filters to refine our search results.

### 2.2 Inclusion criteria

Studies were included if they met the following criteria:

1. Published in peer-reviewed journals in English.
2. Focused on cancer epigenetics, including mechanistic studies, biomarker development, and technological advances,
3. Presented original research, systematic reviews, meta-analyses, or significant technological developments.
4. Provided insights into the latest advancements in the field of cancer epigenetics.
5. Published between January 2018 and August 2024.

### 2.3 Exclusion criteria

Studies were excluded if they met the following criteria:

1. Not directly related to cancer epigenetics.
2. Focused solely on genetic alterations without epigenetic components.
3. Publications that were conference abstracts, editorials, letters, or commentaries without substantial data.

### 2.4 Data extraction and synthesis

Titlezs and abstracts were screened for relevance. Full-text articles from the potentially eligible studies were assessed. Data on study characteristics, methodologies, key findings, and strong implications for cancer research and treatment were extracted. Discrepancies were resolved through discussions and consensus.

The extracted information was synthesized and organized by major themes in cancer epigenetics, including mechanisms, biomarkers, advanced computational methods, and technological advances.

## 3 Overview of epigenetic mechanisms in cancer

Cancer epigenetics is reshaping our understanding of tumor biology. This rapidly evolving field explores how epigenetic alterations drive cancer development and progression, thereby opening new frontiers in precision medicine (12, 19). At its core, cancer epigenetics focuses on a suite of dysregulated processes now recognized as hallmarks of several tumors. These include aberrant DNA methylation, histone modifications, chromatin remodeling, and altered expression of non-coding RNAs and miRNAs. When the cell’s instruction manual is rewritten without permission, the consequences can be profound: tumor suppressor genes can be silenced, whereas oncogenes are activated, fueling cancer growth. As researchers, we decoded this epigenetic language to develop targeted therapies that could outsmart cancer in its own game. **Table 1** provides a snapshot of the main epigenetic mechanisms implicated in cancer and serves as a foundation for understanding this complex and fascinating field.

**Table 1:** Summary of main epigenetic modifications and their effects on gene expression.

Epigenetic Modification	Description	General Effect on Gene Expression
DNA Methylation	Addition of a methyl group to cytosine residues in CpG dinucleotides	Typically represses gene expression when occurring in promoter regions; global hypomethylation can lead to genomic instability
Histone Modifications	Post-translational modifications of histone proteins, including acetylation, methylation, phosphorylation, and ubiquitination	Varies depending on the specific modification: - Acetylation generally activates transcription - Methylation can either activate or repress, depending on the specific amino acid residue and extent of methylation -Phosphorylation plays roles in chromosome condensation during cell division, DNA damage repair, and transcriptional regulation -Ubiquitination involves DNA damage response. Monoubiquitylation of H2A is associated with gene silencing, while H2B ubiquitination correlates with transcription activation
Chromatin Remodeling	Dynamic modification of chromatin architecture through repositioning, ejection, or restructuring of nucleosomes	Can either activate or repress gene expression by altering DNA accessibility to transcription factors and other regulatory proteins
Non-coding RNAs	Functional RNA molecules that are not translated into proteins, including microRNAs (miRNAs) and long non-coding RNAs (lncRNAs)	-Non-coding RNAs (ncRNAs) are generally involved in fine-tuning gene expression, chromatin modification, RNA slicing, protein synthesis, cell differentiation and development, and can act as either activators or repressors through various mechanisms - MicroRNAs (miRNAs) typically inhibit translation or lead to mRNA degradation - (Long non-coding RNAs (lncRNAs) can interact with chromatin-modifying complexes to regulate gene expression Besides the well-known ncRNAs such as Transfer RNAs (tRNAs) and Ribosomal RNAs (rRNAs), there are also several other types of ncRNAs, including: small interfering (siRNAs), Small nuclear RNAs (snRNAs), Small nucleolar RNAs (snoRNAs), Piwi-interacting RNAs (PiRNAs), and Circular RNAs (circRNAs)

### 3.1 DNA methylation

**Figure 1:**
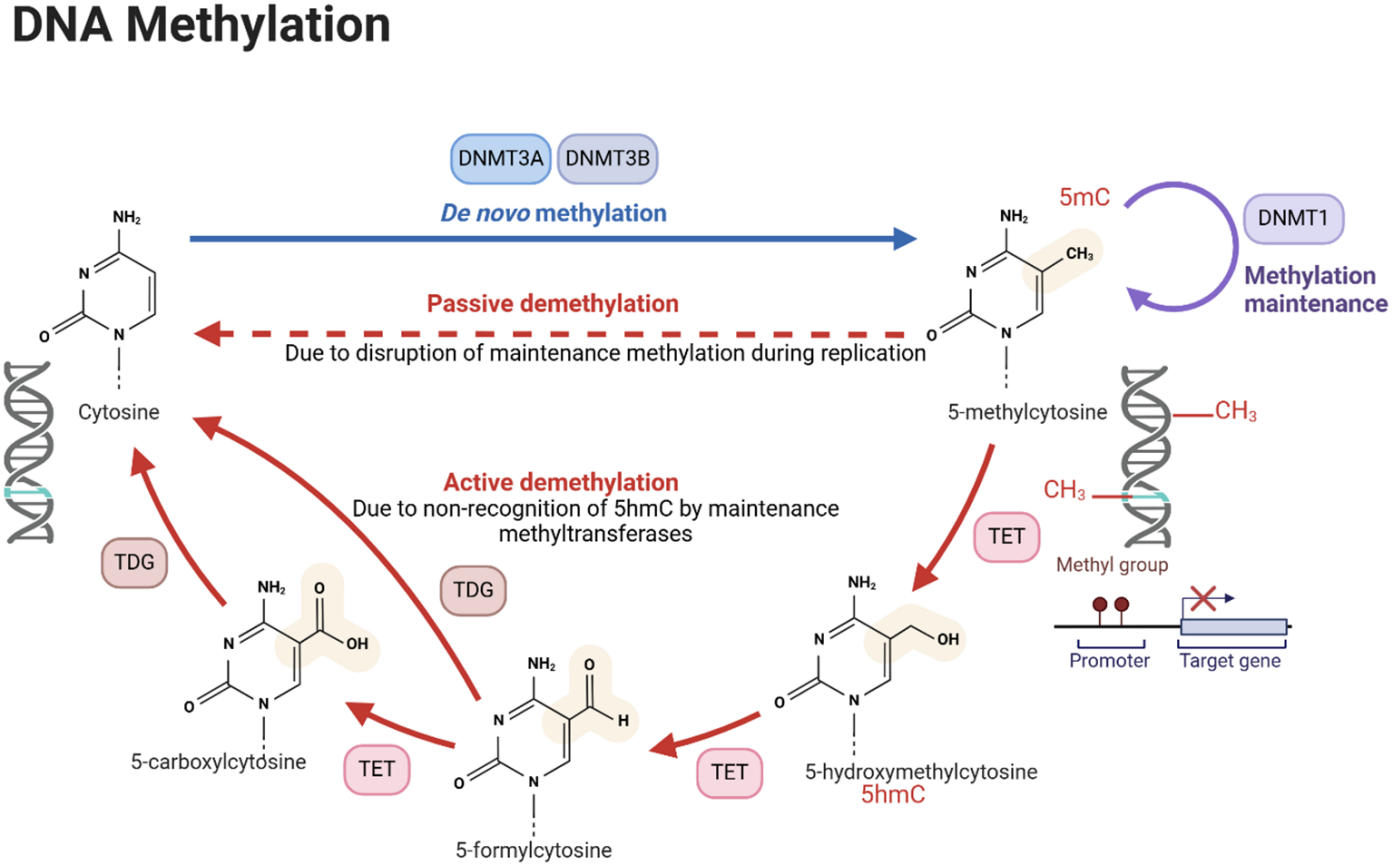
DNA methylation process. The figure illustrates the processes of *de novo* DNA methylation, passive demethylation, and active demethylation. The first section, *de novo* methylation, shows the addition of methyl groups (CH_3_) to cytosine residues in DNA, catalyzed by DNMT DNA methyltransferases (DNMTs), resulting in a newly methylated DNA strand. The second section, passive demethylation, describes the process of DNA replication without incorporating new methyl groups, leading to a gradual reduction in methylation across successive cell divisions. The third section, active demethylation, highlights the enzymatic conversion of 5-methylcytosine (5mC) to 5-hydroxymethylcytosine (5hmC) by ten-eleven translocation (TET) enzymes and TDG, followed by further oxidation steps and replacement of modified bases by unmodified cytosine through the base excision repair pathway. DNA methylation is crucial for the regulation of gene expression, development, and disease. Created using Biorender.

DNA methylation is a well-characterized prima donna of epigenetic modifications that involves the addition of a methyl group to the 5-carbon position of cytosine residues within CpG dinucleotides. DNA methylation is a high-maintenance process that must be maintained to avoid passive and active demethylation cycles during DNA replication. This is because, on the one hand, we have faithful maintenance by methyltransferases, working overtime to preserve these crucial epigenetic signatures. On the other hand, the forces of entropy and active demethylation threaten to erase these hard-won modifications. It is a constant and perpetual battle enacted within the nucleus of every dividing cell (**Figure 1**).

**Figure 2:**
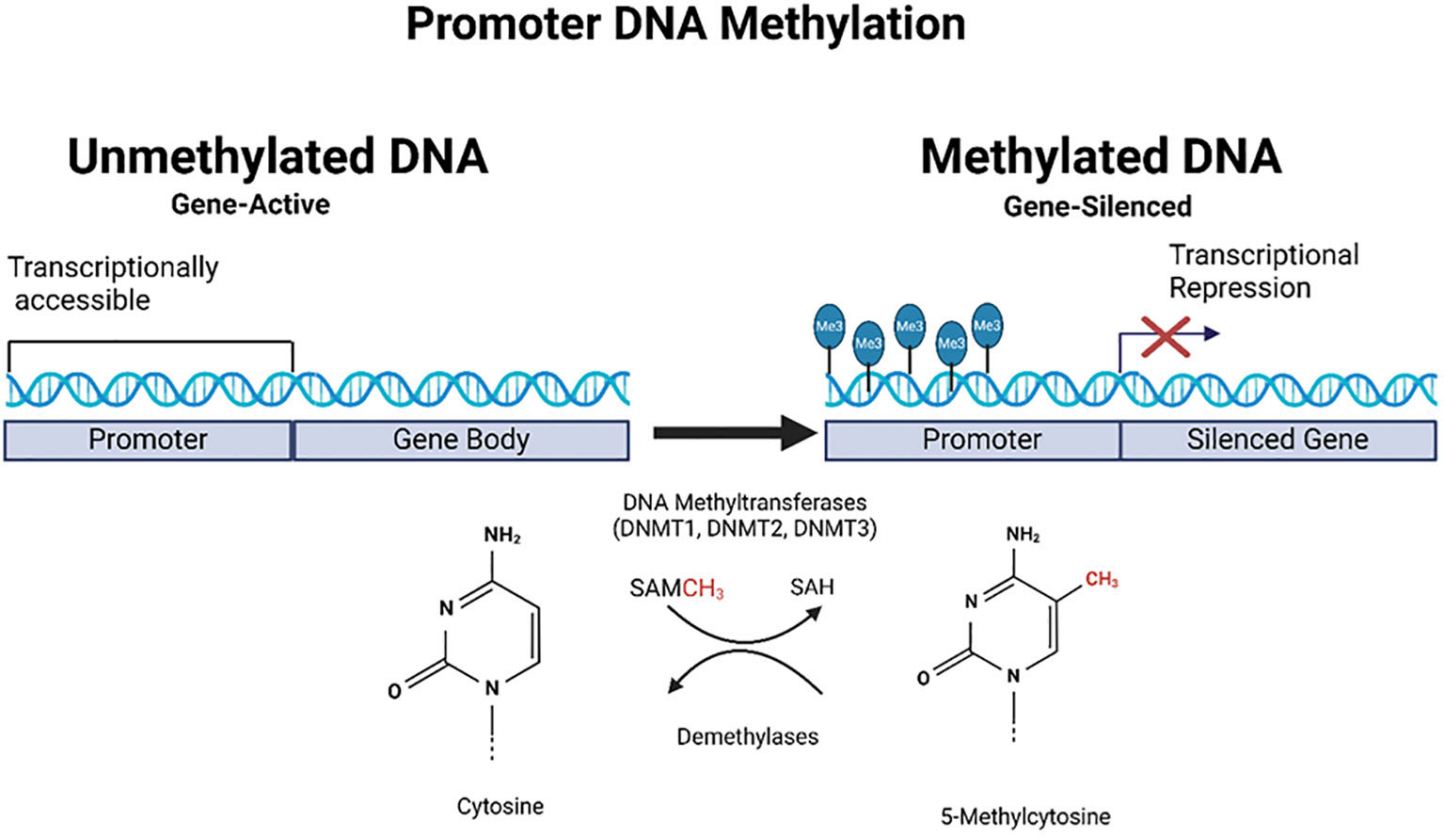
Schematic representation of promoter DNA methylation in gene regulation. This figure illustrates the role of DNA methylation in gene regulation, focusing on promoter regions. On the left, the unmethylated promoter had trimethylation marks on histones, indicating active transcription. On the right, the promoter is methylated, catalyzed by DNA methyltransferases (DNMT1 and DNMT3), and RNA (tRNA) methyltransferase (DNMT2, not shown in the figure) using S-adenosylmethionine (SAM) as a methyl donor. This methylation leads to transcriptional repression, as indicated by blocked transcription, resulting in gene silencing. The chemical structures of cytosine and 5-methylcytosine are shown at the bottom of the illustration, representing bases before and after methylation. Created using Biorender.

These changes are critical in the initiation and progression of various cancers as they disrupt normal gene regulation. Abnormal DNA methylation patterns are commonly found in cancer cases; global hypomethylation can cause instability, whereas hypermethylation of specific promoters often leads to the suppression of tumor suppressor genes (20, 21) (**Figure 2**). It should also be noted that certain DNA methylations such as intragenic DNA methylation (IGM) show distinct patterns in cancer cells, with genome-wide hypomethylation including clustered hypomethylated CpG sites in gene-poor regions, 5’ regions of frequently expressed genes, all occurring alongside specific hypermethylation events at chromosome breakpoints (22). These patterns differ significantly from normal tissue and contribute to genomic instability.

#### 3.1.1 Recent advances in DNA methylation and cancer

Advancements in the field of DNA methylation and its relationship with cancer have recently made headways. Global hypomethylation is a common feature of cancer cells and is associated with genomic instability. This decrease in methylation typically occurs within regions called partially methylated domains (PMDs), which can trigger the activation of oncogenes that aid tumor development (23). These domains are often found in non-CpG regions, such as solo-WCGW sequences near A or C nucleotides (23). For example, collagen triple helix repeat containing-1 (CTHRC1) plays a significant role in modulating cell proliferation and invasion in hepatocellular carcinoma (HCC), potentially through mechanisms related to DNA methylation (21). Research indicates that CTHRC1 is overexpressed in HCC tissues and correlates with poor clinical outcomes, including larger tumor size and increased metastasis (24, 25). The expression of CTHRC1 is influenced by various signaling pathways, including TGF-β and PI3K/AKT, which are known to promote tumor progression and epithelial-mesenchymal transition (EMT) (25, 26). Additionally, studies have shown that DNA methylation can regulate the expression of genes involved in HCC, suggesting that CTHRC1 may also be affected by methylation changes (27, 28). Thus, CTHRC1 not only contributes to HCC cell invasion and proliferation but may also be modulated by epigenetic factors, highlighting its potential as a therapeutic target and prognostic biomarker in HCC management.

The field of DNA methylation research has made significant strides by offering new insights into cancer development, progression, and potential treatments. A notable example of these advancements is the pioneering study that employed cell-free DNA methylation analysis. This innovative approach demonstrated exceptional precision in identifying and pinpointing various types of cancers. The test exhibited an impressive 99.4% specificity and sensitivity ranging from 60% to 94% across 16 distinct cancer types (29, 30).

To further advance this field, researchers have developed MethMarkerDB, a comprehensive database of cancer DNA methylation biomarkers based on whole-genome bisulfite sequencing data. This valuable resource identified an astounding 5.4 million differentially methylated regions across 13 common cancer types, providing researchers with a powerful tool for discovering novel cancer biomarkers (31). Researchers have also developed a new liquid biopsy method using methylation-sensitive restriction enzyme sequencing (MRE-Seq) combined with deep neural network analysis. This approach, termed methPLIER, showed high sensitivity and accuracy in detecting cancer-specific DNA methylation patterns in cell-free DNA, offering potential for early cancer diagnosis (32). This novel DNA methylation analysis tool enables cross-dataset comparative analyses and reduces bias between datasets caused by differences in preprocessing methods and analysis platforms, facilitating integrated analysis across multiple studies (32).

In organ-specific studies, including prostate cancer, researchers have found that hypermethylation of specific genes distinguishes between tumor and normal tissues. Methylation patterns can also differentiate between aggressive subtypes, including neuroendocrine prostate cancer and castration-resistant prostate adenocarcinoma (29). Furthermore, research on CpG island hypermethylation phenotype (CIMP) in prostate cancer has revealed associations with distinct clinical features and outcomes. This knowledge could lead to targeted treatments for CIMP subtypes in prostate cancer (29). A study of head and neck cancer showed that DNA hypermethylation of tumor suppressor genes such as p16, PTEN, DAPK, MGMT, ECAD, and RASSF1A leads to decreased expression of these genes, contributing to cancer development and poor prognosis (31).

Studies on testicular germ cell tumors have indicated that DNA methyltransferase inhibitors (DNMTi) and demethylases (DMT) may serve as potential therapeutic agents. These substances can undo abnormal DNA methylation patterns linked to cancer advancement (23). These developments highlight the potential of DNA methylation analysis to revolutionize early cancer detection and classification methods, offering new avenues for prognosis and targeted therapies.

Although DNA methylation studies have provided valuable insights, the field faces challenges and limitations, including the following:

- Heterogeneity of methylation patterns across different cancer types and even within the same tumor.
- Difficulty in distinguishing driver from passenger methylation changes.
- Complexity in interpreting the functional significance of methylation changes due to their reversibility.
- Technical challenges in accurately measuring methylation at single-cell resolution.

### 3.2 Histone modifications

**Figure 3:**
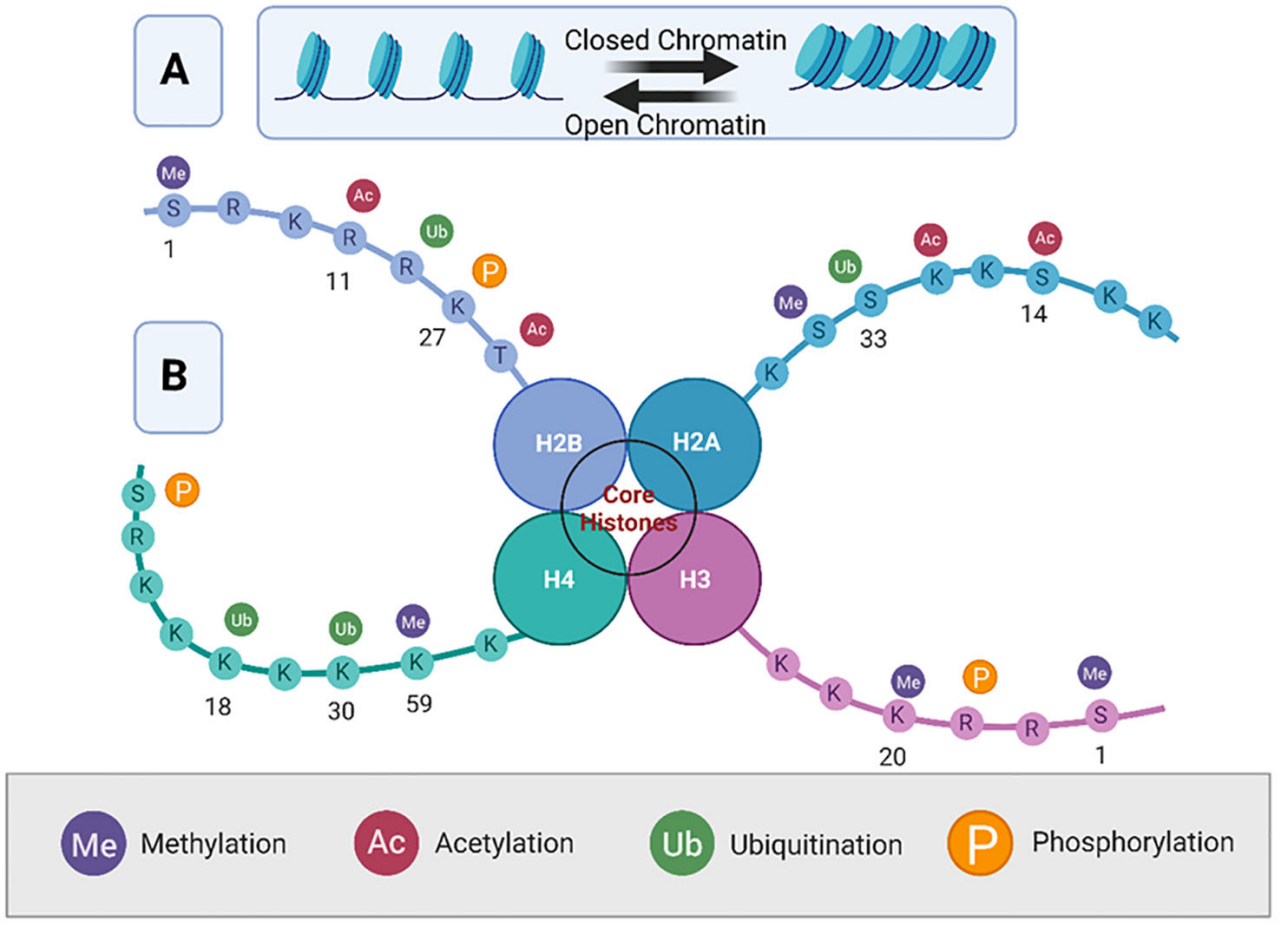
Overview of histone modifications and their roles in chromatin dynamics. This figure represents a simplified octameric structure of the nucleosome core particle. Panel **(A)** illustrates the dynamic equilibrium between open (euchromatin) and closed (, heterochromatin) chromatin. Panel **(B)** illustrates the nucleosome structure of histone proteins H2A, H2B, H3, and H4, showing various post-translational modifications of the histone tails. The four main modifications are methylation (Me), acetylation (Ac), ubiquitination (Ub), and phosphorylation (P), each of which is color-coded. Modifications are indicated on specific amino acid residues (denoted by their single-letter codes) along the histone tails, demonstrating their potential impact on chromatin structure and gene regulation. The specific locations of these modifications are important for understanding their roles in transcriptional regulation, with methylation typically associated with gene repression, and acetylation with gene activation. Created using Biorender.

Histone proteins, around which DNA is wrapped, can undergo various post-translational modifications such as methylation, acetylation, phosphorylation, and ubiquitination (**Figure 3**).

**Figure 4:**
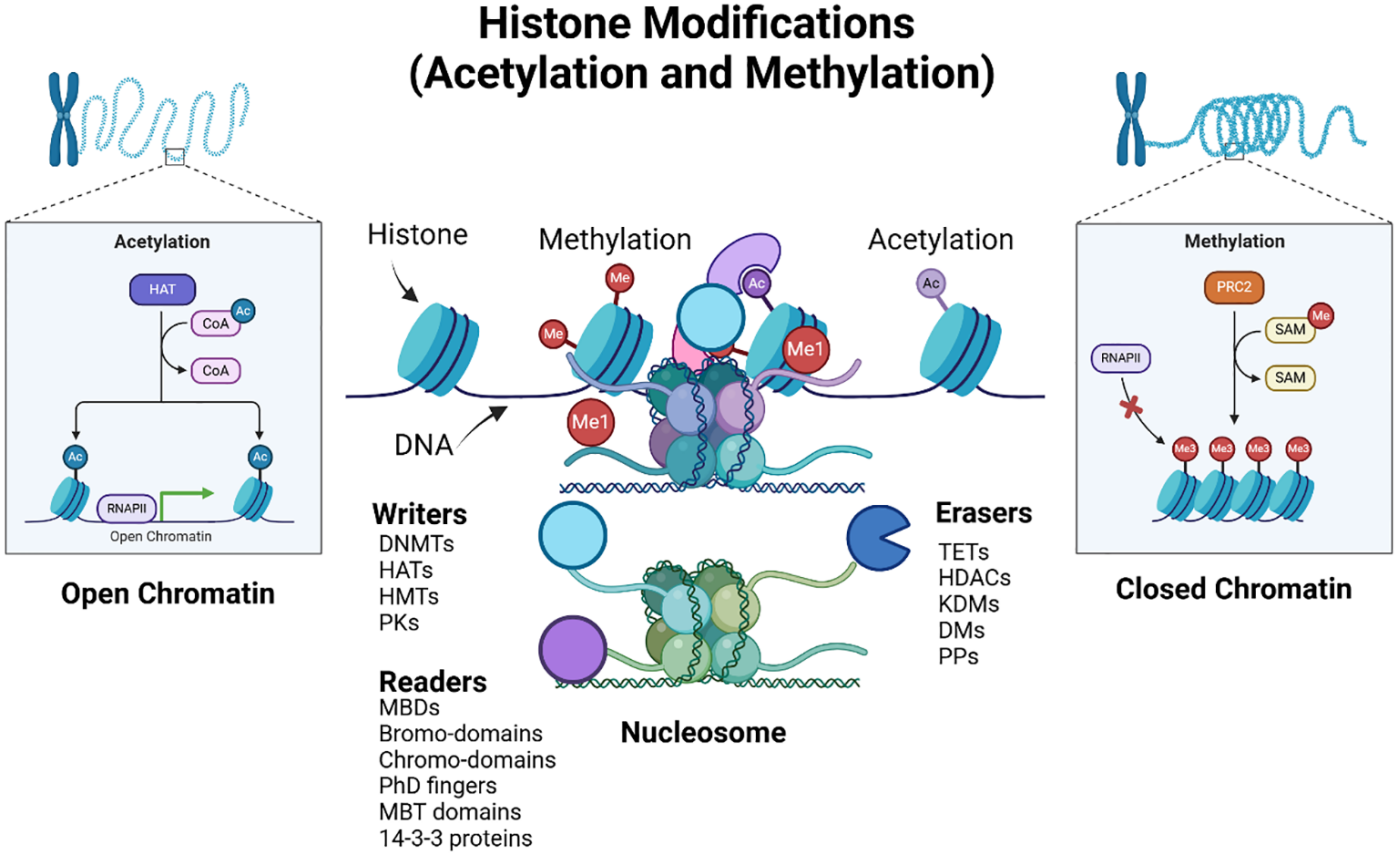
Epigenetic histone modifications. The figure illustrates histone modifications, specifically acetylation (Ac) and methylation (Me), and their effects on the chromatin structure and gene expression. The diagram shows two states of chromatin: open chromatin (euchromatin) and closed chromatin (heterochromatin). In the open chromatin state, histone acetyltransferases (HATs) add acetyl groups to histone tails, facilitating gene expression by allowing RNA polymerase II (RNAPII) access to DNA, whereas histone deacetylases (HDACs) remove them. The inset also details the acetylation process, showing the transfer of acetyl groups (Ac) from acetyl-CoA to histones, resulting in transcriptional activation. In the closed chromatin state, histone methyltransferases (HMTs), such as PR-Set7 and Polycomb Repressive Complex 2 (PRC2), add methyl groups, (while demethylases (DMs) remove them), resulting in transcriptional repression. The inset shows the methylation process, where S-adenosyl methionine (SAM) acts as the methyl donor, causing gene silencing by preventing RNAPII binding. The figure also depicts the nucleosome structure, showing the DNA wrapped around histone proteins. Key epigenetic players are highlighted: Writers (such as DNA methyltransferases (DNMTs), histone acetyltransferases (HATs), HMTs, and protein kinases (PKs) add modifications, readers (including MBDs (Methyl-CpG Binding Domain proteins) bromodomains, chromodomains, plant homeodomain (PhD) fingers, and Malignant Brain Tumor (MBT) domains recognize these modifications, and Erasers TETs (Ten-Eleven Translocation enzymes), HDACs, histone lysine demethylases (KDMs), DMs, and protein phosphatases (PPs)) remove them. The interplay between these factors determines chromatin accessibility and gene expression. Created using Biorender.

These modifications influence the chromatin structure and gene expression (33, 34). In cancer, dysregulation of histone modifications can lead to either the activation of oncogenes or repression of tumor suppressor genes. For instance, histone acetylation generally correlates with transcriptional activation, whereas methylation can either activate or repress transcription depending on the specific amino acid residues modified (33, 35) (**Figure 4**).

#### 3.2.1 Recent advances in histone modification and cancer

Recent advances in histone modification research have significantly enhanced our understanding of cancer biology and its therapeutic strategies. Histone modifications, including methylation, acetylation, phosphorylation, and ubiquitination, play crucial roles in regulating gene expression and chromatin structure, with dysregulation linked to various malignancies, such as acute myeloid leukemia and head and neck squamous cell carcinoma (HNSCC) (36, 37). The interplay of these modifications, orchestrated by “writers,” “erasers,” and “readers,” (see **Figure 4**) has emerged as a critical factor in cancer development, prompting the exploration of co-targeting histone modulators for precision therapy (38–40). Furthermore, specific histone modifications are implicated in telomere dynamics, influencing genomic stability and oncogenic transformation (41, 42).

**Figure 5:**
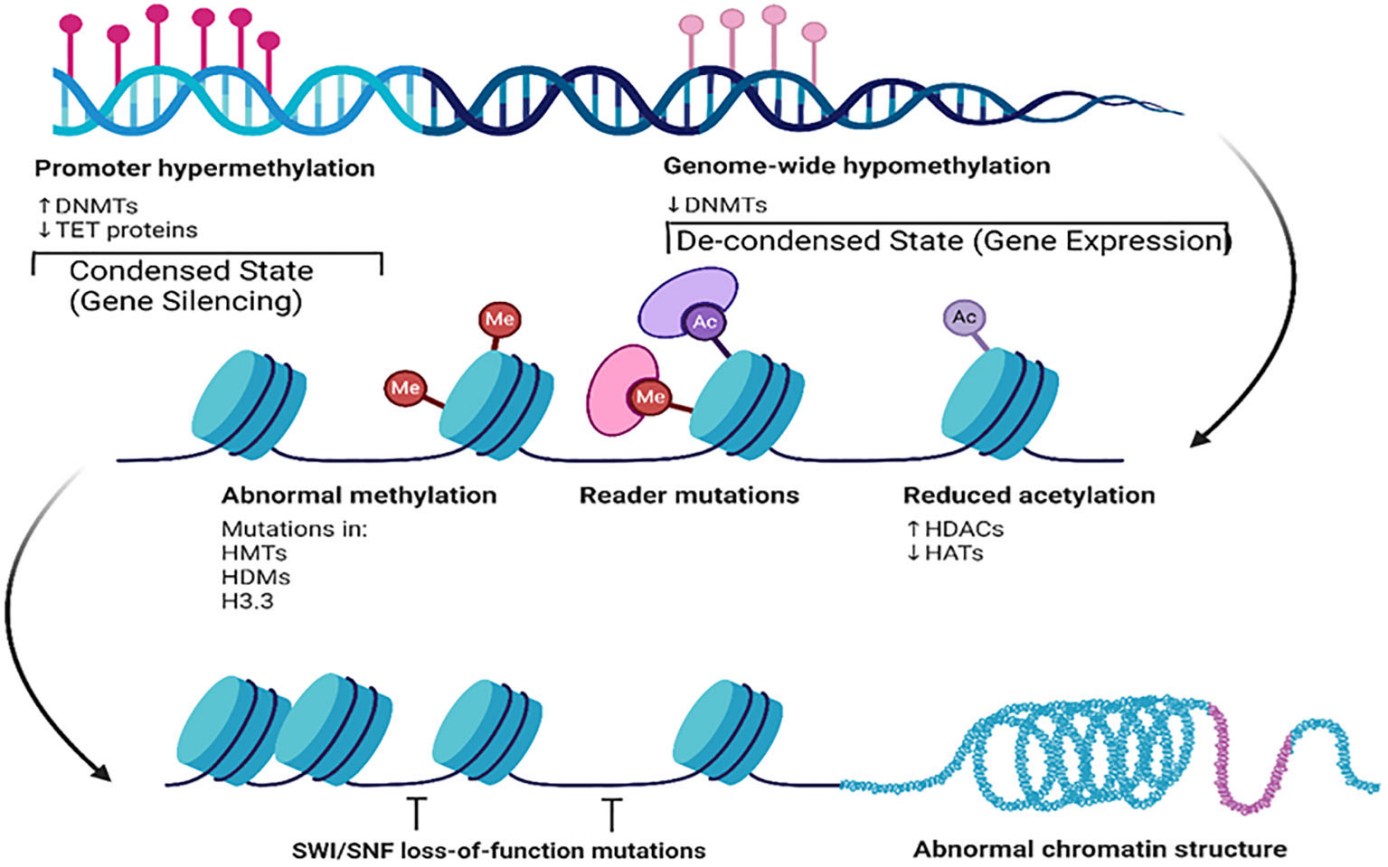
Key epigenetic mechanisms and their effects on chromatin structure and gene expression. This figure illustrates the contrasts with promoter hypermethylation and genome-wide hypomethylation. Promoter hypermethylation, characterized by increased DNMT and decreased TET protein activity, leads to condensed chromatin and gene silencing. Conversely, genome-wide hypomethylation results in a de-condensed state and gene expression. This figure also illustrates abnormal methylation, reader mutations, and reduced acetylation along with their impact on chromatin structure. Additionally, it shows how SWI/SNF loss-of-function mutations affect nucleosome positioning, contributing to abnormal chromatin structures. Created using Biorender.

The development of small-molecule inhibitors targeting histone methyltransferases and other epigenetic regulators represents a promising avenue for cancer treatment, with ongoing clinical trials highlighting the potential of these strategies (36, 37). For instance, researchers have identified a new histone modification, lysine benzoylation (Kbz), that plays a role in transcriptional regulation. The SAGA complex acts as a writer for Kbz, which is associated with active transcription and functions distinctly from other modifications, such as acetylation. It is regulated by sirtuin 2 (SIRT2), which serves as an eraser and removes the benzoyl group, thus influencing gene expression dynamics (43). This modification is elevated in various cancer types, including lung and colorectal cancers, suggesting its potential as a therapeutic target. The presence of Kbz, along with other acylation markers, suggests a complex interplay between metabolism and epigenetic regulation, highlighting its potential as a biomarker for cancer (44). Other recent studies have focused on small-molecule inhibitors of histone H3 lysine 36 (H3K36) methyltransferases, which are implicated in various cancers (45–47). Another recent research uncovered a different relationship between histone methylation and cancer through H3 N-terminal arginine mutations rather than specifically H3K36 methylation (48). These and other histone methylation mechanisms in cancer cells provide insights into the interplay between different epigenetic modifications. Understanding the structure and function of H3K36 methyltransferases is critical for developing cancer treatment strategies. Crosstalk also exists between histone modifications and DNA methylation (**Figure 5**).

Together, the images in **Figures 4**, **5** provide a comprehensive overview of how post-translational epigenetic modifications influence gene regulation and chromatin organization. Overall, the integration of histone modification research into cancer therapy paves the way for innovative therapeutic approaches.

Although histone modification studies have provided valuable insights, the field faces challenges and limitations, including the following:

- The dynamic nature of histone modifications makes them difficult to study in a static context. Histone modifications are highly dynamic and can change rapidly in response to cellular signals. This makes it challenging to capture the full extent of histone modification in each cell or tissue.
- Crosstalk between different histone modifications complicates the interpretation. Histone proteins can undergo multiple modifications at different sites, resulting in complex combinatorial codes that are difficult to interpret.
- Limited understanding of the combinatorial effects of multiple histone modifications.
- Technical challenges in studying histone modifications at specific genomic loci. Furthermore, analyzing histone modifications requires specialized techniques such as chromatin immunoprecipitation (ChIP) sequencing. These methods can be labor-intensive and require careful experimental design to minimize artifacts.

### 3.3 Chromatin remodeling

**Figure 6:**
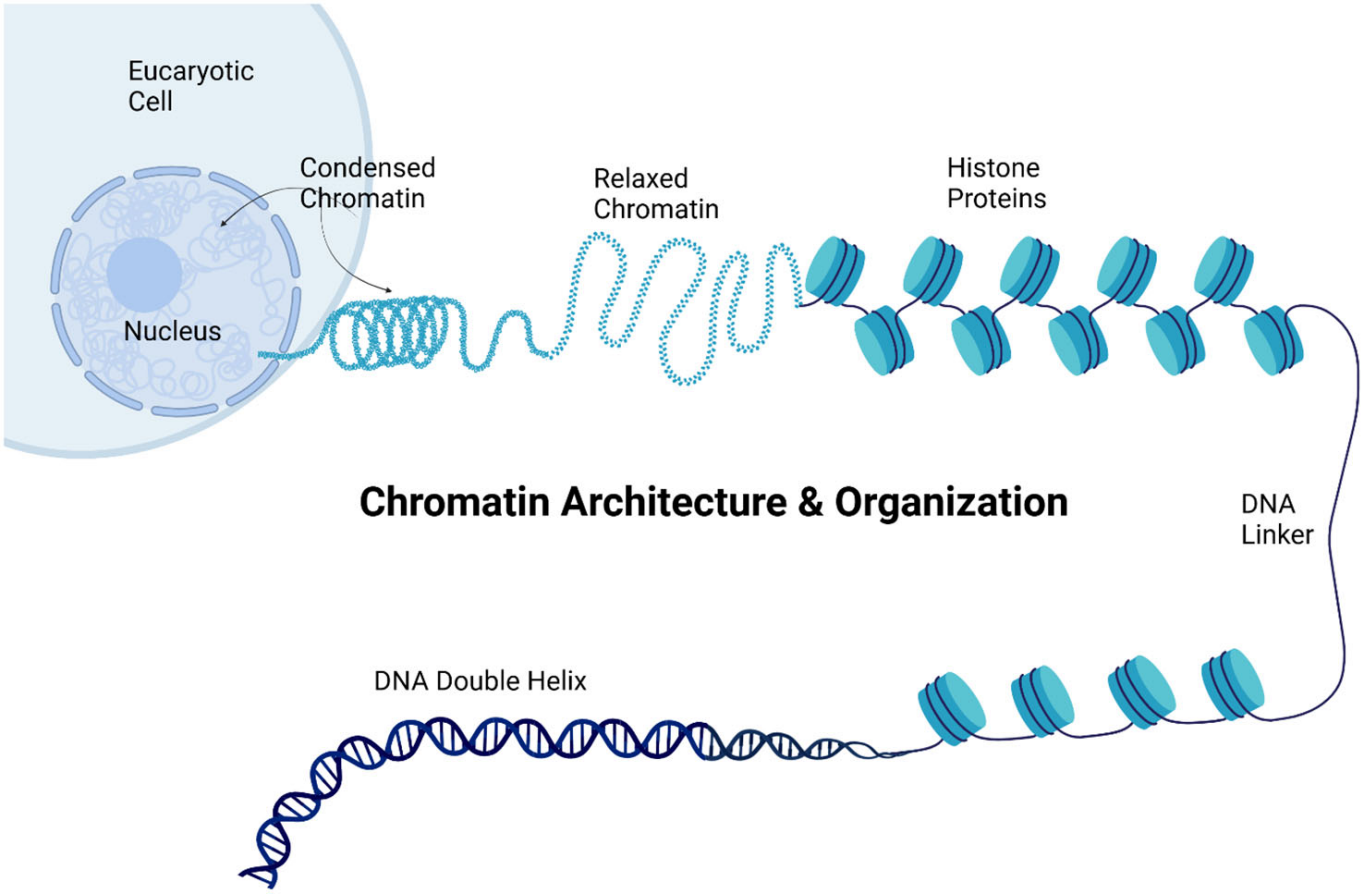
Chromatin organization. The figure illustrates chromatin architecture and organization within a eukaryotic cell, showing the hierarchical structure of the genetic material. The image progresses from the cellular level to the molecular structure of the DNA. At the top, a eukaryotic cell is depicted with its nucleus containing both condensed and relaxed chromatin. Condensed chromatin appears as a tightly coiled structure, representing transcriptionally inactive regions, while relaxed chromatin appears as a looser, more open structure associated with active gene expression. The figure then displays a string of nucleosomes, the basic units of chromatin, consisting of histone proteins around which the DNA is wrapped. The DNA double helix is illustrated at the bottom, representing the most fundamental level of DNA organization. This comprehensive visualization demonstrated how genetic material is packaged and organized within the cell, from the highly condensed chromatin visible at the cellular level to the molecular structure of the DNA double helix, highlighting the importance of this organization in gene regulation and expression. Created using Biorender.

Chromatin remodeling involves the dynamic modification of the chromatin architecture and organization to allow access to the genomic DNA (**Figure 6**).

This process is mediated by chromatin remodeling complexes that reposition, eject, or restructure nucleosomes (34). In cancer, mutations in chromatin remodeling genes can lead to altered chromatin states, affecting gene expression patterns crucial for cell growth and differentiation (49). Chromatin remodeling is crucial for regulating gene expression, particularly in cancer, where mutations in chromatin remodeling genes can lead to altered chromatin states and disrupted cellular functions. Research indicates that chromatin remodeling complexes, which reposition or restructure nucleosomes, are essential for the transition of chromatin from a repressed to an active state, thereby influencing gene activation and cellular differentiation. For example, chromatin remodeling complexes, such as SWI/SNF, are essential for transitioning chromatin from a repressed to an active state, thereby influencing gene activation and cellular differentiation (50, 51). In head and neck squamous cell carcinoma (HNSCC), chromatin remodeling is linked to the differentiation of cancer cells, suggesting that targeted differentiation strategies can mitigate malignancy (52). Similarly, in thyroid cancer, SETMAR facilitates chromatin remodeling, enhances the expression of differentiation-related genes, and affects treatment responses (53). Furthermore, dysregulation of chromatin dynamics, including mutations in remodeling complex genes, has been associated with various cancers, highlighting the importance of these processes in disease progression and potential therapeutic interventions (52, 54). Thus, understanding chromatin remodeling mechanisms is vital for developing effective cancer therapies.

#### 3.3.1 Recent advances in chromatin modification and cancer

Chromatin sequencing (Chrom-seq) is an antibody-free method that combines specific chromatin mark readers with APEX2 enzyme to identify RNAs associated with various chromatin modifications. This technique utilizes proximity biotinylation to label and isolate RNAs near specific histone modifications, enabling the systematic mapping of chromatin-associated RNAs that play regulatory roles in epigenetic events. This process, which has proven more efficient than previous methods in terms of sensitivity and cost, requires fewer cells and less processing time (55).

Role of Epigenetic Readers: Epigenetic readers are proteins that recognize specific chromatin modifications and facilitate gene expression and regulation. For example, Stadler et al., highlighted how chromatin modifications can recruit various reader proteins, indicating a complex interplay between these modifications and gene activity (56). The methyl-CpG-binding domain (MBD) has shown versatility in recognizing non-canonical epigenetic marks, as demonstrated by Kosel et al., suggesting that readers can adapt to different epigenetic contexts (57).

Advances in Chromatin Profiling: Techniques such as SAM-seq allow for the simultaneous profiling of chromatin accessibility and DNA methylation, revealing how these features interact within nucleosomes (58). This integration enhances our understanding of how RNAs and epigenetic markers influence chromatin structure. Another methodology is CUT&RUN (Cleavage Under Targets and Release Using Nuclease) [https://www.epicypher.com/resources/cut-and-run-overview/] (59). This is used for profiling histone modifications and other chromatin-bound proteins, which are key components of epigenetic regulation. CUT&RUN is also used for high-resolution epigenomic mapping of histone modifications and identifying binding patterns for chromatin-associated proteins and transcription factors genome-wide (59). Some of its advantages over ChiP-seq for epigenomic studies include lower input requirements (can be used with as few as 100,000 cells), higher signal-to-noise ratio, and better resolution of binding sites.

In contrast, while the focus on readers provides insights into chromatin regulation, the dynamic nature of chromatin modifications and their interactions with various cellular factors remains a complex area that requires further exploration.

Although chromatin remodeling studies have provided valuable insights, the field faces challenges and limitations, including the following:

- Complexity of chromatin remodeling complexes and their context-dependent functions.
- Difficulty in studying dynamic chromatin changes in real time. Chromatin remodeling complexes are highly dynamic and can be regulated by various factors, making it difficult to understand their precise roles in gene expression.
- Limited understanding of how chromatin remodeling interacts with other epigenetic mechanisms. Chromatin remodeling involves the interplay of multiple proteins and factors, making it difficult to dissect the specific contributions of each component.
- Technical challenges in capturing transient chromatin states. Studying chromatin remodeling complexes requires specialized techniques, such as ChIP-seq and biochemical purification. However, these methods are challenging and time consuming.

### 3.4 Non-coding RNAs

**Figure 7:**
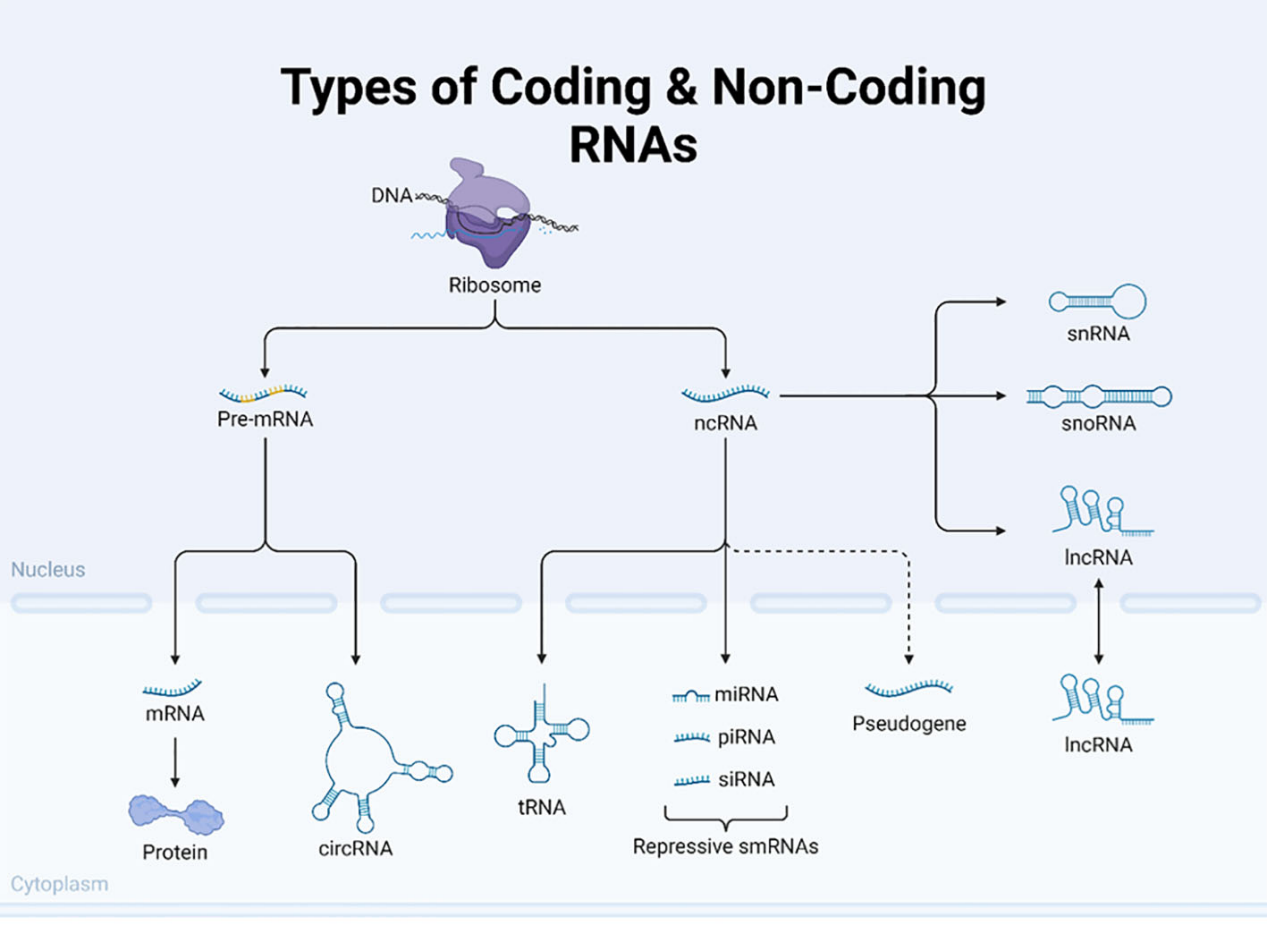
Types of coding and non-coding RNAs. This figure illustrates the diverse types of coding and non-coding RNA molecules in eukaryotic cells. The image depicts the flow from DNA to various RNA species centered around the ribosome. The left side shows the pathway of coding RNA, starting with pre-mRNA in the nucleus, which is processed into mature mRNA and exported to the cytoplasm, where it is translated into protein. The figure also highlights circular RNA (circRNAs) as a unique form of coding RNA. The diagram on the right shows several types of non-coding RNAs (ncRNAs), including transfer RNA (tRNA) and various small RNAs, such as microRNAs (miRNAs), piwi-interacting RNA (piRNAs), and small interfering RNA (siRNAs), collectively labeled as repressive smRNAs. Additionally, it depicts other non-coding RNAs like small nuclear RNA (snRNA), small nucleolar RNA (snoRNA), and long non-coding RNA (lncRNA). The figure also includes pseudogenes, which are nonfunctional gene sequences. By presenting both coding and non-coding RNAs, this illustration effectively demonstrates the complexity and diversity of RNA molecules involved in gene expression and regulation in eukaryotic cells. Created using Biorender.

Non-coding RNAs (ncRNAs), including microRNAs (miRNAs) and long non-coding RNAs (lncRNAs) (see more ncRNAs in **Table 1** and **Figure 7**), play significant roles in the regulation of gene expression at the post-transcriptional level, thereby influencing processes such as cell proliferation, apoptosis, and metastasis in cancer. In fact, the 2024 Nobel Prize in Physiology and Medicine was awarded jointly to Victor Ambros and Gary Ruvkun “for the discovery of microRNA and its role in post-transcriptional gene regulation” [https://www.nobelprize.org/prizes/medicine/2024/press-release/]. In the early 1990s, Ambros and Ruvkun uncovered a novel, evolutionarily conserved mechanism of gene regulation by microRNAs, which revealed an unexpected layer of genetic control, revolutionizing our understanding of molecular biology. MicroRNAs are now being explored for potential diagnostic and therapeutic uses, particularly in cancer and other diseases.

miRNAs can bind to messenger RNAs (mRNAs) and inhibit their translation, leading to degradation or translational inhibition (60). Dysregulation of miRNAs and lncRNAs is commonly observed in cancer, where they can function as oncogenes or tumor suppressors, affecting processes such as cell proliferation, apoptosis, and metastasis (60, 61). These epigenetic mechanisms are not isolated; they often interact to regulate gene expression in a coordinated manner. For example, the lncRNA HOTAIR has been found to promote cervical cancer progression by regulating BCL2 by targeting miR-143-3p (62). miR-34a promotes DNA methylation and histone deacetylation, leading to gene silencing and tumor suppression. Research indicates that miR-34a is often downregulated in various cancers, including non-small cell lung cancer (NSCLC), and its low expression correlates with tumor progression (63, 64). The loss of miR-34a has been shown to promote tumorigenesis, whereas its overexpression can inhibit cancer cell proliferation and invasion (65, 66). Furthermore, miR-34a’s role in regulating immune responses within the tumor microenvironment underscores its multifaceted anti-tumor effects (65). In contrast, lncRNAs can interact with chromatin-modifying complexes to regulate gene expression. For example, lncRNA MALAT1 (metastasis-associated lung adenocarcinoma transcript 1) has been shown to promote chromatin compaction and gene silencing, contributing to cancer progression in several ways, and MALAT1 has been found to interact with chromatin remodeling subunits such as BRG1, a component of the SWI/SNF complex. This interaction promotes inflammation-related hepatocellular carcinoma progression by epigenetically regulating the expression of inflammatory genes, such as IL-6 and CXCL8 (67). MALAT1 can also recruit PRC2 components such as EZH2 to specific genomic loci, leading to H3K27 trimethylation and subsequent gene silencing. This mechanism has been observed in colorectal cancer and other malignancies (68, 69).

Overall, ncRNAs have emerged as promising biomarkers for cancer detection, as they can be easily detected in the blood, urine, and other bodily fluids. They are often more sensitive and specific than traditional cancer markers (70, 71). Understanding these biomarkers is crucial to unravel the complexities of cancer biology and develop effective therapeutic strategies. Future research may increasingly delve into the mechanisms of action of messenger RNA and circular RNA, aiming to develop targeted treatment strategies utilizing non-coding RNA drugs.

#### 3.4.1 Recent advances in non-coding RNAs and cancer

Recent advances in non-coding RNAs (ncRNAs) have significantly enhanced our understanding of cancer biology, particularly tumor progression, drug resistance, and therapeutic potential. These findings underscore the dual role of ncRNAs as both oncogenes and tumor suppressors, paving the way for novel cancer diagnostics and treatments.

Role of Long Non-coding RNAs (lncRNAs): lncRNAs are crucial in cancer pathophysiology, influencing tumorigenesis and metastasis through various mechanisms, including transcriptional regulation and histone modification (72). They are involved in the regulation of key metabolic pathways such as glucose metabolism by modulating the expression of glucose transporters such as GLUT1, which is often overexpressed in cancer cells (72). This entails that they can act as biomarkers for cancer therapeutics, with many still awaiting identification (73, 74). They also regulate drug resistance mechanisms, such as ATP transporter overexpression and epithelial-mesenchymal transition, highlighting their potential to overcome treatment challenges (75).

ncRNAs in Tumor Microenvironment: Advances in RNA sequencing have revealed the significant role of ncRNAs in the tumor microenvironment, suggesting their potential as therapeutic targets (76). Specific ncRNAs have been implicated in gynecologic cancers, acting as either oncogenic or tumor-suppressive agents, with ongoing clinical trials exploring their utility as biomarkers (77).

Emerging Insights on Circular RNAs: Circular RNAs (circRNAs) are gaining attention for their regulatory roles in cancer, although they remain less characterized than lncRNAs and microRNAs (78).

Although advancements in ncRNA research offer promising avenues for cancer treatment, challenges and limitations remain in fully understanding their complex roles and mechanisms in various cancers.

- Vast number of non-coding RNAs with unknown functions. In addition, ncRNAs can function as both oncogenes and tumor suppressors, and their roles can vary depending on the cellular context. Understanding the complex regulatory networks involving ncRNAs is an ongoing challenge.
- Difficulty in predicting and validating ncRNA targets.
- Biological complexity in understanding tissue-specific roles of non-coding RNAs. The expression and function of ncRNAs can vary significantly between different tissues and cancer types, making it difficult to identify universally applicable biomarkers.
- Technical challenges in detecting and quantifying low-abundance ncRNAs. Identifying and characterizing ncRNAs can be challenging because of their diverse sizes and functions. High-throughput sequencing technologies have improved our ability to study ncRNAs; however, challenges remain in terms of data analysis and interpretation.

## 4 Advanced technologies for epigenetic profiling

This review highlights the cutting-edge techniques and advances in epigenetic profiling technologies that have emerged in recent years.

### 4.1 Single-cell epigenomics

**Figure 8:**
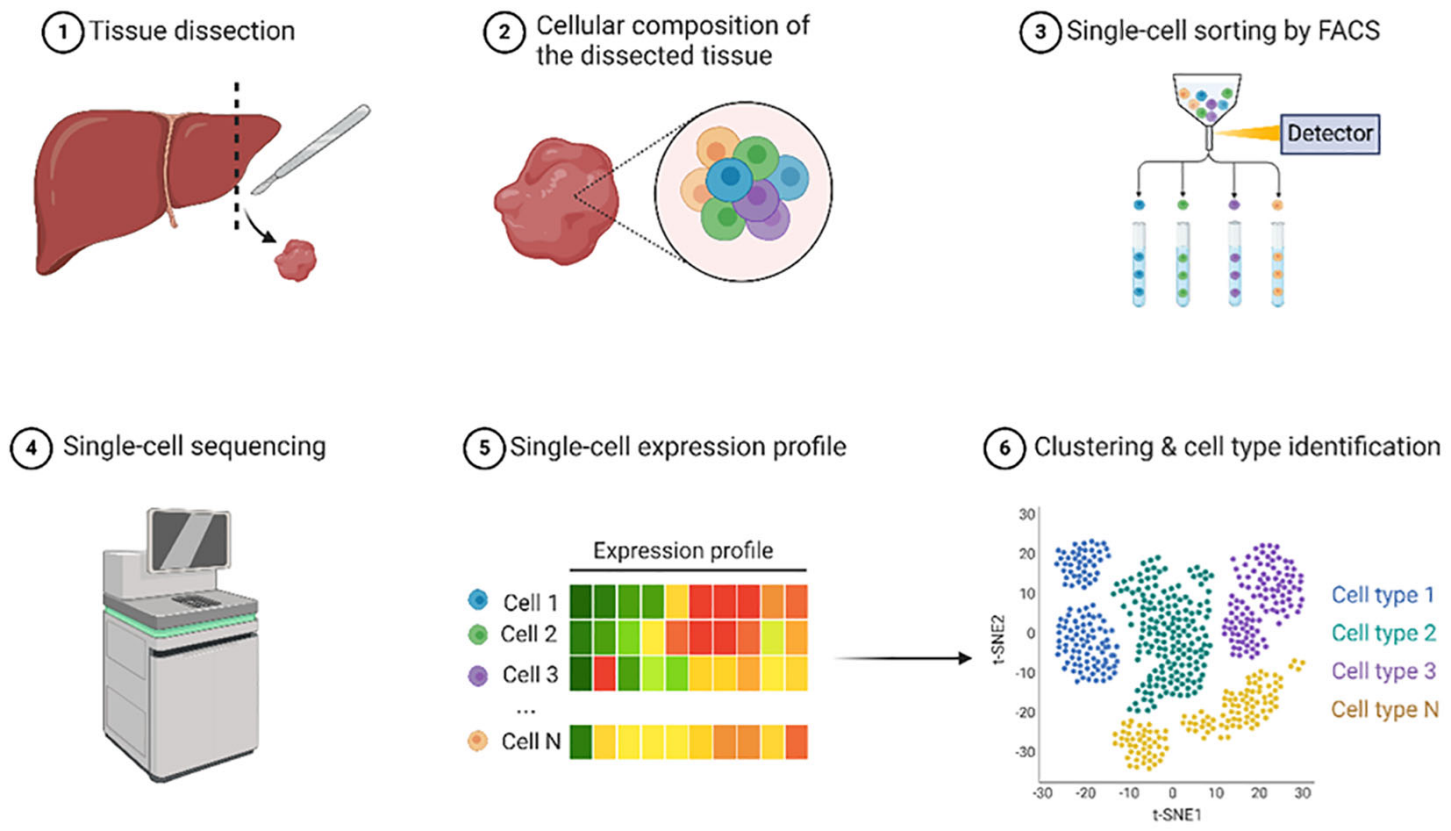
Single-cell sequencing. This illustration outlines the process of single-cell epigenomic analysis focusing on liver tissue. The workflow begins with tissue dissection where a liver sample is obtained. The cellular composition of the dissected tissue was examined, revealing diverse cell types represented by circles of different colors. Next, single-cell sorting was performed using Fluorescence-Activated Cell Sorting (FACS) to separate individual cells into distinct tubes. Sorted cells were subjected to single-cell sequencing using specialized equipment. From these sequencing data, single-cell expression profiles were generated and visualized as colored bars indicating the gene expression levels for each cell. Finally, the expression data were used for clustering and cell type identification, resulting in a t-SNE plot, where different colors represent distinct cell populations. This comprehensive workflow demonstrates how individual cells from a tissue sample can be isolated, analyzed at the single-cell level, and characterized to identify various cell types within the original tissue. Created using Biorender.

Decoding the biological complexity from individual cells to integrated tissues requires comprehensive single-cell profiling techniques. DNA methylation is crucial for regulating gene expression and cellular functions at the individual cell level. Scientists can uncover intricate details regarding cellular heterogeneity, developmental processes, and disease mechanisms by examining DNA methylation patterns in single cells. Recent advancements in single-cell sequencing technologies have revolutionized the study of epigenetic heterogeneity within tumors. Single-cell epigenomics allows the profiling of DNA methylation, chromatin accessibility, and histone modifications at the single-cell level, providing insights into the diverse epigenetic landscapes of cancer cells and their microenvironment. Epigenetic sequencing is the use of high-throughput sequencing technology to quantify and analyze DNA modifications involved in gene expression and regulation of cell differentiation and development. The stepwise process, from tissue dissection to single-cell sequencing, is shown in **Figure 8**.

Single-Cell ATAC-seq (scATAC-seq): Single-cell ATAC-seq (scATAC-seq) has significantly advanced our understanding of chromatin accessibility at the individual cell level, revealing cell type-specific regulatory landscapes in tumors (79). This technique allows for the mapping of open chromatin regions in individual cells, revealing cell type-specific regulatory landscapes in tumors. Recent improvements and innovations such as scifi-ATAC-seq and txci-ATAC-seq have notably increased throughput, allowing for the indexing of up to 200,000 nuclei in a single reaction, which is approximately a 20-fold increase compared to traditional methods, and has increased throughput and reduced input requirements (80, 81). These advancements not only enhance the efficiency of data collection but also reduce the input requirements, making the technique more accessible for diverse applications. Furthermore, methods such as SANGO improve cell annotation by integrating genomic sequences with scATAC-seq data, thereby addressing challenges related to high dimensionality and sparsity (82). Collectively, these improvements facilitate a more comprehensive exploration of the regulatory landscapes in tumors, enabling the identification of both known and unknown cell types, which is crucial for understanding tumor heterogeneity and identifying potential therapeutic targets.

Single-cell DNA Methylation Sequencing: Methods such as scBS-seq and snmC-seq2 provide high-resolution DNA methylation profiles of individual cells, uncovering epigenetic heterogeneity within tumors (83). These techniques are pivotal in elucidating epigenetic heterogeneity within tumors. These methods also allow for high-resolution profiling of DNA methylation at the individual cell level, revealing cell-specific epigenetic changes that are crucial for understanding tumor biology and heterogeneity. For instance, the scBS-seq method has been shown to effectively identify rare cell populations and improve differential methylation analysis despite the challenges posed by sparse data and zeros in sequencing results (84). Additionally, advancements in single-cell sequencing technologies have enhanced our understanding of epigenetic mechanisms that contribute to tumor heterogeneity, capture diverse omics layers, and provide insights into intratumoral variations (85). Furthermore, novel techniques such as Cabernet enable high-throughput methylome profiling, facilitating the analysis of complex tissues, including tumors, at a single-cell resolution (86). Collectively, these methodologies underscore the importance of single-cell sequencing in uncovering the intricate epigenetic landscape of tumors.

Single-cell multi-omics approaches such as scNMT-seq (single cell nucleosome, methylation, and transcription sequencing) simultaneously profile DNA methylation, chromatin accessibility, and gene expression in single cells, offering a comprehensive view of the epigenetic state by simultaneously profiling DNA methylation, chromatin accessibility, and gene expression within individual cells. This approach has provided unprecedented insights into epigenetic heterogeneity and its impact on gene regulation in cancer (87). Moreover, techniques such as ISSAAC-seq, which can be implemented in both plate-based and droplet formats, allow for the interrogation of chromatin accessibility and gene expression in the same nucleus, achieving high data quality with approximately 10,000 ATAC reads and 2,000-5,000 detected genes per cell (88–90). The integration of multimodal omics data enhances our understanding of cellular processes, revealing complex interactions and regulatory networks that are often obscured in bulk analyses (88). Furthermore, recent advancements in integrating single-cell multimodal epigenomic data using convolutional have demonstrated the potential for improved analysis of diverse epigenomic modalities, facilitating a unified representation of cellular states (91). Collectively, these techniques underscore the transformative impact of single-cell multi-omics in elucidating the intricate molecular landscape of cells.

Although single-cell epigenomic studies have provided valuable insights, the field faces challenges and limitations, including the following:

- High cost and technical complexity of single-cell sequencing technologies. approaches can be expensive and require specialized equipment and expertise. This limits their widespread adoption and application in clinical settings.
- Data sparsity and noise in single-cell epigenomic data. The analysis of large-scale single-cell datasets requires advanced computational tools and bioinformatics expertise. This can be a bottleneck for researchers, particularly those with limited computational resources.
- Computational challenges in integrating and analyzing multimodal single-cell data.
- Biological interpretation of the results of single-cell and multi-omics studies can be challenging because these data can reveal complex patterns and relationships that are difficult to understand.
- Limited throughput compared to bulk sequencing approaches.

### 4.2 Multi-omics approaches

**Figure 9:**
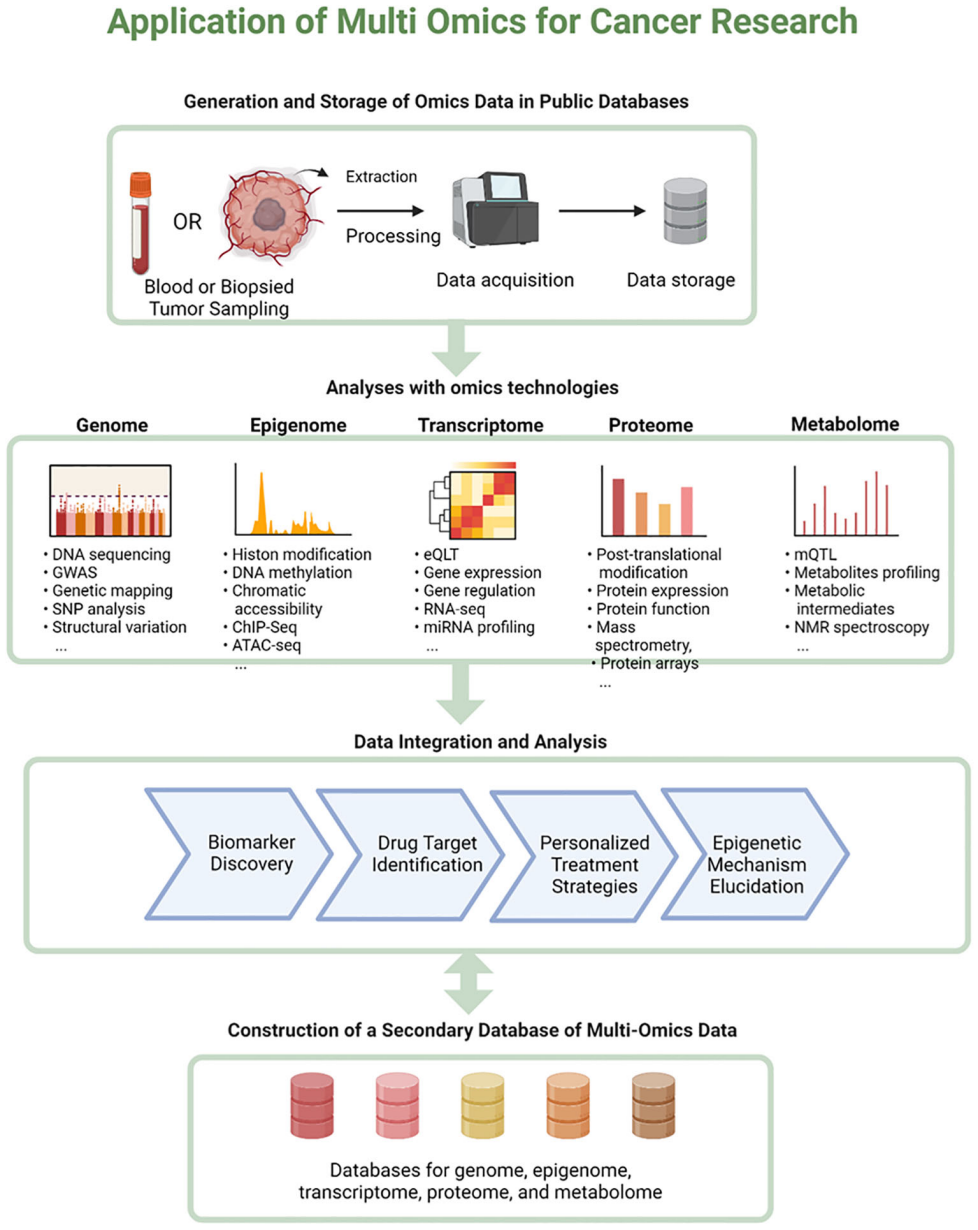
Application of multi-omics for cancer research. This figure illustrates the application of multi-omics approaches in cancer research. The flowchart begins with the generation and storage of omics data in public databases, showing the process from sample collection (blood or biopsied tumor) to data acquisition and storage. It then branches into five main omics technologies: the Genome, Epigenome, Transcriptome, Proteome, and Metabolome. Each branch lists specific techniques and data types associated with that field, such as DNA sequencing for genomics, and metabolite profiling for metabolomics. The next step shows data integration and analysis, leading to outcomes like biomarker discovery, drug target identification, personalized treatment strategies, and epigenetic mechanism elucidation. The flowchart concludes with the construction of a secondary database of multi-omics data encompassing databases for genome, epigenome, transcriptome, proteome, and metabolome information. This comprehensive diagram effectively illustrates the workflow and potential application of integrative omics in cancer research. Created using Biorender.

A holistic approach to cancer research drives transformative progress by providing a comprehensive understanding of the disease. A popular application of next-generation sequencing (NGS) is epigenomic profiling, which provides a mechanistic context for genome regulation in cancer. By integrating multi-omics data, including genomics, transcriptomics, proteomics, and epigenomics, researchers have uncovered intricate regulatory networks within cancer cells. This deeper understanding facilitates the discovery of novel epigenetic biomarkers and therapeutic targets, ultimately leading to improved cancer prevention, diagnosis, and treatment (92–94). For instance, studies have demonstrated that integrative machine learning techniques can manage the heterogeneity of multi-omics datasets, leading to significant insights into cancer mechanisms and discovery of potential biomarkers (93, 95). Additionally, analyses of datasets such as TCGA-BRCA have uncovered key hub genes and enriched pathways relevant to breast cancer, further illustrating the potential of multi-omics for identifying therapeutic targets (96). Moreover, comprehensive multi-omics frameworks allow the exploration of shared biological processes in tumorigenesis, thereby prioritizing drug development (95, 97). Overall, the integration of diverse omics data is essential for advancing precision medicine in oncology (98–101) (**Figure 9**).

Integrated Multi-omics Platforms: Advanced integrated platforms have been developed to simultaneously analyze multiple omics data types. For instance, the MOFA+ (Multi-omics Factor Analysis Plus) framework, an advanced statistical tool designed for the integration of multimodal single-cell data, allows for the integration of epigenomic data with other omics layers, revealing hidden biological factors driving tumor heterogeneity (102–104). Integrated multi-omics platforms, such as the MOFA framework, are pivotal for elucidating the complexities of tumor heterogeneity by combining various omics data types, including epigenomic, transcriptomic, and proteomic information. For instance, the Flexynesis tool enhances usability in precision oncology by integrating diverse molecular datasets through deep learning, allowing for unsupervised feature selection and model evaluation, which can reveal hidden biological factors influencing tumor behavior (105). Additionally, the integration of multimodal data, as emphasized in the MINDS framework, is crucial for the development of personalized treatment strategies in oncology (106). The application of deep learning in analyzing complex cancer biology further supports the identification of predictive biomarkers and therapeutic responses, thereby enhancing clinical decision-making (107–109). Overall, Flexynesis represents a pivotal advancement in making deep learning-based multi-omics integration more accessible, thereby fostering improved outcomes in precision oncology (105, 109).

Similarly, the Directional Pathway Mapping (DPM) method is pivotal for integrating omics data as it emphasizes the directional relationships among genes and pathways, thereby enhancing the understanding of tumor dynamics. The DPM facilitates the identification of consistent gene and pathway regulation across various datasets by prioritizing those that exhibit stable changes while penalizing inconsistent data, which is crucial for accurate biological interpretation (110). This method has been effectively applied to analyze multi-omics datasets, such as transcriptomic, proteomic, and DNA methylation data, revealing candidate biomarkers with prognostic significance in cancer (110). Furthermore, the integration of diverse data types, as demonstrated in other studies, underscores the importance of holistic approaches in cancer research, allowing for a comprehensive understanding of the complex interactions that drive tumor progression (111, 112). Overall, the DPM represents a robust framework for elucidating the intricate dynamics of cancer biology through the directional integration of multi-omics data.

Finally, the Multi-omics Analysis Sandbox Toolkit is a significant advancement in the field of multi-omics research that facilitates the exploration, integration, and visualization of diverse multi-omics data, promoting collaborative research efforts and aiding in the identification of potential biomarkers. This online platform allows researchers to connect clinical data with omics information, enhances collaborative efforts, and enables the identification of potential biomarkers through versatile analysis options and visualization outputs (113). Additionally, tools like playOmics, which offers a robust framework for data management and biomarker identification, emphasizing interpretability and model-driven predictions, thus facilitating user engagement and scientific collaboration, and MultiOmicsAgent (MOAgent), an open-source tool that significantly enhances biomarker discovery through its user-friendly interfaces and advanced data analysis capabilities, particularly utilizing machine learning techniques, further support biomarker discovery by providing user-friendly interfaces and advanced data analysis capabilities, including machine learning techniques for handling complex datasets (114, 115). The integration of various omics data types, such as genomics, transcriptomics, and proteomics, enables a more comprehensive understanding of diseases, such as cancer, which is crucial for identifying reliable biomarkers and improving diagnostic accuracy. Overall, these tools collectively promote transparency, reproducibility, and collaboration in multi-omics research, ultimately aiding the advancement of precision medicine.

Collectively, these platforms underscore the importance of integrated analyses in uncovering the intricate biological mechanisms underlying tumor heterogeneity, ultimately contributing to improved clinical outcomes.

Single-Cell Multi-omics: Single-cell multi-omics techniques, such as scNMT-seq, are powerful multi-omics methods that enable simultaneous profiling of three molecular layers in single cells: Use GpC methyltransferase to label open chromatin; combine bisulfite sequencing for DNA methylation analysis; and integrate RNA sequencing for transcriptome profiling (87, 92). For instance, the scCancerExplorer database integrates over 110 datasets from various cancer types, allowing researchers to explore gene expression, DNA methylation, and chromatin accessibility, thereby providing insights into cancer biology and patient outcomes (116). Furthermore, combining single-cell gene expression analysis (RNA-Seq) with single-cell open chromatin mapping (ATAC-Seq) provides genome-wide mapping of both the transcriptome and epigenetic landscapes at the single-cell level, allowing the identification of how epigenetic changes affect gene expression in distinct cell populations. Additionally, single-cell multi-omics studies have demonstrated the essential roles of transcription factors in regulating gene expression through chromatin interactions, highlighting the complexity of gene regulatory networks in cancer (117). However, challenges remain in effectively integrating multimodal data, which can hinder the discovery of regulatory associations (117). Overall, these techniques provide unprecedented insights into the epigenetic landscape of cancer and underscore their potential for therapeutic advancement.

Proteogenomics in Cancer Epigenetics: Proteogenomic approaches combine genomic, transcriptomic, and proteomic data to provide a more complete picture of how epigenetic changes affect protein expression and function in cancer. Recent studies have used this approach to identify novel epigenetically regulated protein targets in breast cancer (118, 119). For instance, proteomic approaches have significantly advanced our understanding of epigenetic regulation in breast cancer by integrating genomic, transcriptomic, and proteomic data (120). Recent studies have highlighted the utility of these methods in identifying novel epigenetically regulated protein targets that may play critical roles in cancer progression and treatment. Moreover, the development of accessible proteogenomic informatics resources has facilitated the identification of variant protein sequences linked to cancer, thereby enhancing the ability to discover potential therapeutic targets (121). Additionally, the interdisciplinary nature of proteogenomics allows for comprehensive tumor analysis, aiding the discovery of new cancer antigens for immunotherapy. Recent studies have characterized the immune landscape of various tumors, revealing distinct immune subtypes and potential therapeutic targets that are crucial for developing effective immunotherapy strategies (118). The integration of mass spectrometry with proteogenomics further enhances the identification of HLA-bound peptides, which are essential for T-cell recognition in immunotherapy (122). However, the computational complexity of these approaches poses challenges for their widespread adoption among non-expert researchers, which may limit their application in clinical settings (121). Overall, proteogenomics represents a promising frontier in cancer research, particularly for elucidating the functional implications of epigenetic changes in breast cancer.

Metabolo-Epigenomics: Recent advances in integrating metabolomics and epigenomics have revealed crucial insights into cancer biology. Studies show that metabolic programming, a hallmark of cancer, significantly influences epigenetic modifications like DNA methylation and histone alterations, which in turn affect tumor growth and progression (123–126). For example, a recent study identified specific metabolites that drive epigenetic reprogramming in glioblastoma, opening new avenues for therapeutic intervention (127). Other recent studies have highlighted that metabolic reprogramming provides insights into the biochemical pathways involved in cancer, facilitating the identification of novel biomarkers and therapeutic strategies (128–133). The integration of these ‘omics’ approaches allows for a comprehensive understanding of the interplay between metabolism and epigenetics, paving the way for innovative cancer treatments that target these metabolic and epigenetic changes (112, 134–136). This multi-omics strategy enhances the classification of cancer subtypes and elucidates the mechanisms underlying therapeutic resistance and disease progression, as highlighted in recent studies of breast cancer and myelodysplastic syndrome (MDS) (137). Thus, the convergence of metabolomics and epigenomics offers promising avenues for therapeutic interventions in cancer.

Temporal Multi-omics: Time-series multi-omics experiments have been employed to study the dynamic interplay between epigenetic changes and other molecular events during cancer progression and treatment response. A landmark study used this approach to map the temporal order of epigenetic and transcriptomic changes during the development of drug resistance in lung cancer (112, 138). This study highlights the dynamic interplay between various molecular events, revealing that specific mutations and epigenetic modifications could serve as biomarkers for predicting treatment outcomes (139). Moreover, the integration of multi-omics data, including genomics, transcriptomics, and epigenomics, allows for a comprehensive analysis of tumor heterogeneity and the identification of context-specific biomarkers, which are crucial for precision medicine (140). The findings of these studies underscore the importance of temporal multi-omics in mapping the evolution of cancer and tailoring therapeutic strategies, ultimately enhancing patient outcomes (94, 141).

Microbiome-Epigenome Interactions: Emerging research is exploring the interplay between the microbiome and cancer epigenome. Multi-omics approaches integrating microbiome data with host epigenomics have revealed how microbial metabolites can influence epigenetic states in colorectal cancer (CRC) (142, 143). Microbial metabolites, such as short-chain fatty acids (SCFAs) like sodium butyrate, have been shown to induce epigenetic modifications, including histone modifications and DNA methylation, which can influence gene expression and cellular behavior in CRC (144). Additionally, studies indicate that the gut microbiota can program host DNA methylation by affecting methyl donor metabolism, revealing substantial alterations in the DNA methylome of CRC tissues compared to adjacent normal tissues (20). Multi-omics approaches integrating microbiome and metabolome data have further elucidated distinct host-microbiome associations, suggesting that microbial-derived metabolites may serve as potential therapeutic targets. These metabolite-mediated interactions require deeper mechanistic studies to develop targeted CRC interventions (145). Overall, these findings underscore the potential of microbiome-epigenome interactions in shaping CRC pathogenesis and therapeutic strategies.

AI and Machine Learning in Multi-omics Integration: Advanced machine learning algorithms, particularly deep learning models, have been developed to integrate and interpret complex multi-omics datasets. These tools have been successful in predicting cancer outcomes and identifying novel epigenetic biomarkers by leveraging the complementary information from different omics layers (146, 147). For instance, a review highlighted the successful application of machine learning in managing the complexity of multi-omics data, which has led to the identification of critical molecular interactions and potential biomarkers for various cancers (148). In addition, AI-driven frameworks have been proposed to predict causal relationships in biological systems, further emphasizing the potential of these technologies in personalized medicine (149). However, challenges remain, including the need for improved algorithm interpretability and integration of diverse data types, which are essential for clinical applications (150, 151). Overall, the integration of AI and machine learning in multi-omics paves the way for groundbreaking advancements in precision medicine.

Spatial Multi-omics: The development of spatial multi-omics technologies has allowed researchers to study the epigenetic landscape in the context of tissue architecture. These multi-omics approaches have provided unprecedented insights into the complex interplay between epigenetic mechanisms and other molecular processes in cancer. They not only advance our understanding of cancer biology, but also pave the way for more precise and personalized epigenetic therapies.

The limitations and challenges of multi-omics approaches include the following.

- Complexity in integrating diverse data types with different scales and distributions. Heterogeneous omics data types (e.g., genomics, transcriptomics, and epigenomics) have varying levels of noise, biases, and measurement scales. The integration of these diverse data streams requires sophisticated statistical and computational methods.
- High computational requirements and costs for analyzing large multi-omics datasets.
- Difficulty in interpreting the biological variability and significance of the multi-omics integration results. Ensuring consistency across different omics platforms and experimental conditions is crucial for accurate integration of data. This often involves normalization and standardization procedures, which can be complex and time consuming.
- Obtaining sufficient biological samples with high-quality data is often challenging, particularly for rare diseases or specific cell types.
- Limited availability of matched multi-omics datasets for many cancers.

### 4.3 Spatial epigenomics

Spatial epigenomics refers to the study of how epigenetic modifications are spatially organized within the genome, and how this organization influences gene expression and cellular function. It plays a crucial role in understanding cancer biology because epigenetic alterations can lead to tumor progression and metastasis. For instance, chromatin remodeling factors such as lymphoid-specific helicase (LSH) are implicated in regulating DNA methylation patterns, which are essential for maintaining cellular identity and function (72). Spatial epigenomic techniques preserve the spatial context of epigenetic marks within tissue samples, which is crucial for understanding tumor microenvironments. Some of the latest methods related to spatially resolved epigenome mapping include Slide-seq, which has been adapted for epigenetic profiling.

Slide-seqV2: This method improves upon the original Slide-seq by enhancing RNA capture efficiency, allowing for near-cellular-resolution spatial transcriptomics. While primarily focused on transcriptomics, the advancements in Slide-seqV2 have set the stage for its adaptation to other omics, including epigenomics. This method combines advancements in library generation, bead synthesis, and array indexing, resulting in an RNA capture efficiency of approximately 50% of that seen in single-cell RNA sequencing (scRNA-seq), which is approximately ten times greater than that of the original Slide-seq (152). Slide-seqV2 is particularly useful for identifying dendritically localized mRNAs in neurons and characterizing the spatiotemporal development of tissues, such as the mouse neocortex, by integrating spatial information with single-cell trajectory analysis tools (152). Its high transcript detection efficiency and near-cellular resolution make it applicable across various experimental contexts, potentially paving the way for its adaptation to other omics fields, including epigenomics.

Spatial Epigenome Sequencing: This study on Spatial Epigenome Sequencing presents a novel method for spatial epigenome profiling by integrating in-tissue CUT&Tag chemistry with microfluidic deterministic barcoding. This approach allows for spatially resolved genome-wide profiling of histone modifications within tissue sections, specifically targeting modifications, such as H3K27me3, H3K4me3, and H3K27ac. This method provides insights into tissue-specific epigenetic regulation by revealing spatial chromatin states in mouse embryos or olfactory bulbs. It offers the ability to extract single-cell epigenomes *in situ*, thereby enabling the study of epigenetic regulation, cell function, and fate decisions in both normal physiology and pathogenesis (153).

Spatially Resolved Epigenomic Profiling: This approach uses *in situ* tagmentation and transcription, followed by multiplexed imaging to profile histone modifications at single-cell resolution. It provides a high-resolution spatial atlas of epigenetic modifications in tissues, advancing our understanding of the spatial regulation of gene expression. The process begins with *in situ* tagmentation and transcription, which involves direct tagging and transcription of DNA within intact tissue samples (154). This method preserves the spatial context and cellular architecture of the tissues. Following this, multiplexed imaging techniques are used to visualize and quantify histone modifications across individual cells within a tissue section (155). As a result, this technique provides detailed information at the single-cell level, enabling the study of cell-specific epigenetic landscapes and their influence on gene expression. The generation of a comprehensive map of histone modifications helps elucidate the spatial organization of epigenetic marks in complex tissues. By linking spatial epigenetic information with gene expression data, researchers can gain insights into how epigenetic modifications influence cellular functions and tissue development. Furthermore, this method can be applied to investigate spatial epigenetic changes associated with diseases, potentially leading to the identification of novel therapeutic targets. Overall, Spatially Resolved Epigenomic Profiling represents a significant advancement in the field of epigenomics, providing a powerful tool for exploring the intricate relationship between epigenetic modifications and gene expression in a spatially resolved manner.

Spatial Epigenome–Transcriptome Co-profiling: This cutting-edge technology enables the simultaneous profiling of chromatin accessibility and gene expression, histone modifications, and gene expression at a near-single-cell resolution. This method provides valuable insights into how epigenetic mechanisms control the transcriptional phenotypes and cell dynamics within tissues. By linking the epigenome to the transcriptome pixel-by-pixel, researchers can uncover new insights into spatial epigenetic priming, differentiation, and gene regulation within the tissue architecture. This technology has been applied to various tissues, including embryonic and juvenile mouse brains, and adult human brains, revealing both concordant and distinct patterns of tissue features, suggesting differential roles in defining cell states (153).

Spatial Omics Sequencing: This method refers to the development and application of spatial omics techniques, including Slide-seq and Spatial-CUT and Tag, for high-resolution mapping of chromatin accessibility and histone modifications, highlighting their significance in understanding tissue development and disease mechanisms.

### 4.4 Long-read sequencing for epigenetic analysis

Long-read sequencing technologies offer new possibilities for the study of complex epigenetic patterns and structural variations.

Nanopore Sequencing for the Direct Detection of DNA Modifications: Nanopore sequencing has emerged as a powerful tool for the direct detection of DNA modifications, circumventing the need for bisulfite conversion, which is traditionally required for methylation analysis. This sequencing technique also allows for the simultaneous analysis of genetic and epigenetic information, thereby enhancing our understanding of gene regulation (156, 157). Recent studies have demonstrated that nanopore sequencing can effectively identify various DNA modifications, including methylation patterns, associated with developmental disorders, thereby providing insights into the underlying pathogenic mechanisms (158). Additionally, advancements in nanopore technology have improved its throughput and accuracy, facilitating the detection of multiple RNA modifications in a single sample, and underscoring its versatility in epi-transcriptomic research (159, 160). Overall, nanopore sequencing represents a significant advancement in genomic technologies, enabling comprehensive analyses of both genetic and epigenetic landscapes in a single workflow.

PacBio Sequencing for Long-Range Epigenetic Patterns: PacBio’s HiFi sequencing has been used to study long-range epigenetic patterns and their association with chromatin structure in cancer genomes (161, 162). PacBio’s HiFi sequencing technology has proven to be instrumental in elucidating long-range epigenetic patterns and their relationship with chromatin structure, particularly in cancer genomes. For instance, recent studies have used PacBio long-read sequencing to generate detailed chromatin maps, revealing nucleosome footprints and nucleosome-depleted regions, which are critical for understanding chromatin dynamics at the single-molecule level (163, 164). This approach allows for the identification of chromatin heterogeneity, which is essential in cancer research as it can reflect the variability in chromatin structure associated with tumorigenesis. Moreover, the ability of PacBio sequencing to capture long fragments of circulating cell-free DNA (cfDNA) has been linked to altered fragmentation patterns in various cancers, suggesting a connection between the chromatin structure and cfDNA characteristics (165). These findings highlight the potential of PacBio HiFi sequencing not only to map epigenetic modifications but also to provide insights into the chromatin landscape in cancer, thereby enhancing our understanding of its role in disease progression.

Spatial epigenomics, a relatively new approach, has provided researchers with another tool to decipher and understand the core of cancer epigenetics. Spatial epigenomics offers significant potential for unraveling the spatial organization of epigenetic modifications and their roles in tissue development, disease, and other biological processes. However, there are limitations and challenges to its utility, including the following.

- Technical challenges in preserving spatial information while obtaining high-resolution epigenomic data. Current spatial transcriptomic technologies have limitations in spatial resolution, making it difficult to precisely localize epigenetic modifications within individual cells or subcellular compartments.
- Complex tissues with high cellular density and heterogeneity can pose challenges for accurate spatial localization. Additionally, the interpretation of spatial epigenetic patterns can be influenced by the surrounding cellular environment and tissue context.
- Limited throughput and high cost of current spatial technologies.
- Computational challenges in analyzing and visualizing spatial epigenomic data.
- Difficulty in integrating spatial epigenomics with other spatial omics data types. Integrating spatial transcriptomics data with epigenetic profiling data requires sophisticated computational tools and methods to handle the high-dimensional and complex nature of the data.

There are also limitations and challenges associated with Long-read Sequencing. These include:

- Higher error rates compared to short-read sequencing.
- Higher cost per base compared to short-read sequencing.
- Computational challenges in analyzing long-read data, especially for complex genomic regions.
- Limited throughput compared to short-read sequencing.

### 4.5 CRISPR-based epigenome editing and screening

CRISPR technology has been adapted for precise epigenome editing, manipulation, and high-throughput screening of epigenetic regulators, allowing researchers to modify specific epigenetic markers at targeted genomic loci. This approach provides a tool for functional studies of epigenetic modifications and holds potential for therapeutic applications by reactivating silenced tumor suppressor genes.

CRISPR-Cas9 Epigenome Editing Tools: Engineered CRISPR systems, such as dCas9 fused with epigenetic modifiers, allow targeted manipulation of epigenetic marks at specific genomic loci (166, 167). Engineered CRISPR systems, particularly dCas9 fused with epigenetic modifiers, have emerged as powerful tools for the targeted manipulation of epigenetic marks at specific genomic loci. For instance, the dCas9-PPAD system enables site-specific histone citrullination, allowing precise regulation of gene expression by modifying histone marks at targeted loci and demonstrating sustained and specific epigenetic effects (168). Similarly, the dCas9-Tet1 system facilitates targeted demethylation of DNA, providing a method to manipulate DNA methylation at specific sites, thereby influencing gene expression without altering the DNA sequence (169) (169). These systems highlight the potential of CRISPR technology in epigenome editing, as they can fine-tune gene regulation in various cellular contexts, which is crucial for understanding gene expression mechanisms and developing therapeutic strategies (170–172). However, the efficiency of these tools can be influenced by chromatin state, indicating that their effectiveness may vary depending on the local epigenetic landscape (173). Overall, these advancements underscore the versatility and precision of the CRISPR-based epigenome editing tools.

High-Throughput CRISPR Screens for Epigenetic Regulators: Pooled CRISPR screens targeting epigenetic modifiers have identified novel epigenetic vulnerabilities in various cancer types (174). For instance, a study utilizing CRISPR interference (CRISPRi) screens on epigenetic-related genes revealed that inhibiting specific genes significantly affects the differentiation of embryonic stem cells into primordial germ cell-like cells, highlighting the role of epigenetic modifiers in developmental processes (175). Additionally, the integration of CRISPR-ChIP technology has allowed researchers to explore the dynamics of histone modifications, uncovering key regulators involved in oncogenic processes such as the interaction between DOT1L and MLL-AF9 (176). Furthermore, extensive CRISPR knockout screens have identified shared vulnerabilities in chemoresistance across different cancer types, suggesting that targeting epigenetic modifiers could be a strategic approach to overcome therapeutic resistance (176, 177). Collectively, these findings underscore the potential of CRISPR-based methodologies to elucidate epigenetic vulnerabilities in cancer.

However, CRISPR-based epigenome editing and screening tools present several key challenges and limitations.

- Off-target effects: Although Cas9 has been engineered for improved specificity, off-target effects can still occur, leading to unintended epigenetic modifications. CRISPR-based epigenome editing tools can have accidental effects on non-target genomic regions, which may complicate the interpretation of the results and potentially lead to unwanted cellular changes.
- Efficiency and specificity: The efficiency of these tools can be influenced by chromatin state, indicating that their effectiveness may vary depending on the local epigenetic landscape. This variability makes it challenging to achieve consistent results across different genomic regions or cell types.
- Delivery methods: Efficient delivery of CRISPR components to target cells, particularly in vivo, remains a significant challenge. This limitation can affect the applicability of these tools in certain experimental and therapeutic contexts.
- Temporal control: Achieving precise temporal control over epigenetic modifications can be difficult, which may limit the study of dynamic epigenetic processes.
- Multiplexing limitations: CRISPR systems allow for some degree of multiplexing; simultaneously targeting multiple epigenetic marks or genomic loci can be challenging and may lead to decreased efficiency or increased off-target effects.
- Context-dependent effects: The effect of epigenetic modifications can be highly context-dependent, making it difficult to predict the functional outcomes of epigenome editing across different cell types or physiological conditions.
- Long-term stability: Ensuring the long-term stability of induced epigenetic changes, particularly in dividing cells, can be challenging and may require repeated treatments or additional strategies to maintain the desired epigenetic state.
- Complexity of epigenetic regulation: The intricate interplay between different epigenetic markers and regulatory elements makes it challenging to fully understand and control the consequences of targeted epigenetic modifications. Furthermore, epigenetic modifications are often interconnected and influenced by multiple factors. Manipulating one epigenetic modification can have unintended consequences on others.
- Limited understanding of epigenetic writers and erasers: Although CRISPR-based tools can target specific genomic loci, our understanding of how to precisely manipulate the activity of epigenetic writers and erasers is still evolving.
- CRISPR-based epigenome editing tools can have unintended effects on non-target genomic regions, which may complicate the interpretation of the results and potentially lead to unwanted cellular changes. The design of guide RNAs can influence their specificity and off-target effects. Careful design and validation of guide RNAs are crucial for minimizing unintended consequences.
- Screening large libraries of guide RNAs or epigenetic effector domains can be time-consuming and resource-intensive.
- Ethical considerations: The potential for heritable epigenetic changes raises ethical questions, particularly regarding therapeutic applications.

### 4.6 Advanced computational methods

The increasing complexity of epigenomic data has driven the development of sophisticated computational methods. This rapidly evolving area is crucial for interpreting vast amounts of complex data generated by modern epigenomic studies. These methods are essential for extracting meaningful insights from large-scale multidimensional epigenomic datasets in cancer research.

Machine Learning and AI for Epigenetic Data Analysis: Deep learning models have been developed to predict epigenetic states and their impact on gene regulation in cancer (178). Convolutional neural networks (CNNs) and recurrent neural networks (RNNs) have been used to predict epigenetic states using DNA sequence data. For example, DeepCpG uses a CNN-RNN architecture to predict single-cell DNA methylation states, revealing cell type-specific epigenetic signatures in cancer (179). Similarly, pre-trained models on large epigenomic datasets were fine-tuned for specific cancer types, improving the prediction accuracy for smaller datasets. This approach has been successful in predicting enhancer-promoter interactions in rare cancer types (180). Moreover, new techniques such as attention mechanisms and layer-wise relevance propagation are being employed to make AI models more interpretable, helping researchers to understand the features driving epigenetic predictions in cancer (181).

Integrative Multi-Omics Data Analysis Platforms: New computational frameworks enable the integration of epigenomic data with other omics data types, providing a holistic view of cancer biology (148). Novel graph-based approaches have also been developed to integrate diverse epigenomic data types (94, 182). These methods can reveal complex relationships between different epigenetic markers and their impact on gene regulation in cancer (182). Furthermore, multidimensional data integration techniques, such as tensor factorization, are being used to analyze complex epigenomic datasets across multiple cancer types and identify pan-cancer epigenetic signatures (183). Recent advancements in integrative multi-omics data analysis platforms have significantly enhanced our understanding of cancer biology by enabling the integration of diverse omics data types, including epigenomic, genomic, and transcriptomic information. Novel computational frameworks, such as the Multi-omics Imaging Integration Toolset (MIIT), facilitate the spatial integration of these datasets, allowing for a more comprehensive analysis of tumor heterogeneity and gene regulation mechanisms in cancer (184). However, challenges remain, including the need for standardized analytical pipelines and interdisciplinary collaboration to fully leverage these innovative methods in clinical settings (185). Overall, these integrative approaches are promising for refining cancer diagnostic and therapeutic strategies.

Advanced Statistical Methods: Sophisticated Bayesian models were employed to infer causal relationships between epigenetic modifications and gene expression in cancer, accounting for the inherent uncertainty in biological data. Additionally, time-series analysis and new statistical methods for analyzing longitudinal epigenomic data have provided insights into epigenetic dynamics during cancer progression and treatment response. For instance, Bayesian regression frameworks have been developed to analyze the impact of DNA methylation on gene expression, revealing significant epigenetic subnetworks that correlate with cancer progression and patient survival outcomes (186). Additionally, innovative approaches such as trans-dimensional Markov chain Monte Carlo methods enhance the identification of differentially methylated cytosines, thereby addressing the challenges inherent in high-throughput sequencing data (187). Time-series analysis and longitudinal data methods further contribute to the understanding of epigenetic dynamics during cancer treatment, allowing researchers to track changes over time (188). These sophisticated statistical techniques not only improve the accuracy of cancer research, but also facilitate the integration of multi-omics data, paving the way for personalized medicine (189). Overall, the application of these advanced methods is crucial for advancing our understanding of cancer biology and for improving therapeutic strategies.

Network-Based Approaches: Advanced algorithms are being developed to construct and analyze epigenetic regulatory networks in cancer, thereby revealing key hub regulators and potential therapeutic targets (190). Computational approaches that integrate molecular-level epigenetic data with tissue-level information are emerging, providing a more comprehensive understanding of how epigenetic changes influence tumor behavior (191). For instance, regarding Gene Regulatory Network Inference, researchers have developed algorithms like SPIDER the Signaling Protein Interaction Dynamic Extractor and Regulator (SPIDER) to construct gene regulatory networks that incorporate epigenetic data (192). SPIDER uses DNase-seq data to identify open chromatin regions and integrates this information with transcription factor-binding motifs to infer regulatory relationships. This approach has been successful in identifying cell-line-specific regulatory interactions and novel transcription factor-binding events that lack sequence motifs. Another network-based approach involves multiscale network Modeling. A study performed a systematic analysis of co-expression networks across 32 cancer types (193). They identified 4,749 prognostic modules regulated by interactions between gene expression and DNA methylation. The study revealed that co-regulated genes within network modules were enriched in specific chromosome cytobands and preferentially localized in open chromatin regions. This multiscale approach provides insights into epigenetic regulation of cancer prognosis across different cancer types. There is also a network-based approach to epigenetic biomarker identification. Researchers have developed network-based methods to identify epigenetic biomarkers associated with cancer progression (194). For example, a study on esophageal squamous cell carcinoma (ESCC) used a disease-specific gene regulatory network to prioritize CpG sites where methylation changes were most associated with disease progression and patient survival. This approach led to the identification of eight CpG sites located in the promoters of genes such as JAK3, PAX6, E2F5, and CD81 that were significantly correlated with patient survival. In addition, emerging computational methods are attempting to bridge the gap between molecular-level epigenetic data and tissue-level information. For example, researchers are developing approaches to integrate single-cell epigenomic data with spatial transcriptomics, allowing for a more detailed understanding of how epigenetic changes influence tumor heterogeneity and behavior within the tissue context (85).

Cloud-based Platforms and Big Data Analytics: Scalable computing solutions involving cloud-based platforms such as Google Cloud and Amazon Web Services are leveraged to address the massive computational requirements of epigenomic data analysis in cancer research. The integration of distributed computing frameworks like Apache Spark is crucial for efficiently processing large-scale epigenomic and genomic datasets across computer clusters, enhancing both accuracy and computational efficiency in applications such as cancer classification (195, 196). For instance, the ISB Cancer Genomics Cloud (ISB-CGC) facilitates access to large datasets, such as The Cancer Genome Atlas, allowing researchers to employ various programming languages and workflows to efficiently analyze terabytes of genomic data (197). The Seven Bridges Cancer Genomics Cloud further exemplifies this trend by providing secure, on-demand access to data and over 200 bioinformatics tools, enabling scalable and reproducible analyses across global research communities (198). Collectively, these platforms represent a transformative shift in handling complex cancer genomic data, underscoring the importance of cloud computing in modern biomedical research.

Artificial Intelligence for Epigenetic Drug Discovery: Artificial Intelligence (AI) significantly enhances epigenetic drug discovery through advanced target identification and compound screening techniques. Machine learning models are utilized to predict potential epigenetic drug targets by integrating epigenomic data with drug sensitivity information, which allows for more precise identification of therapeutic candidates (199). Additionally, in silico screening methods employing deep learning algorithms are being developed to conduct virtual screening of compound libraries, effectively identifying potential epigenetic modulators (151, 200–202). These AI-driven approaches not only expedite the drug discovery process but also improve the accuracy of predictions of drug interactions and efficacy (203, 204). However, challenges such as data quality and ethical considerations remain, necessitating robust validation frameworks and interdisciplinary collaboration to fully harness AI’s potential in transforming pharmaceutical research (203, 205, 206). Overall, the integration of AI into epigenetic drug discovery represents a promising frontier in the development of novel therapeutics. AI-driven target identification: Machine learning models are employed to predict potential epigenetic drug targets by integrating epigenomic data with drug sensitivity information (207). In silico screening utilizing deep learning models trained on epigenomic data has been used for virtual screening of compound libraries to identify potential epigenetic modulators (208).

Single-cell Computational Methods: Advanced algorithms for inferring cellular trajectories from single-cell epigenomic data reveal the dynamics of epigenetic changes during cancer evolution (209). These algorithms and new computational methods for deconvoluting bulk epigenomic data using single-cell reference datasets have improved our understanding of tumor heterogeneity (210).

These advanced computational methods not only enable researchers to extract more meaningful insights from complex epigenomic datasets, but also drive discoveries in cancer epigenetics. These are essential for translating the wealth of epigenomic data into clinically actionable knowledge. For example, chromatin velocity is a method developed to infer dynamic regulatory landscapes and cellular trajectories from single-cell ATAC-seq data. It models the rate of change in chromatin accessibility to predict future cell states (211). Other currently available monoomic methods for epigenetic single-cell sequencing and spatial epigenomics include ScRRBS for chronic lymphocytic leukemia (212), ScBS-seq for circulating tumor cells (213), and acChip-seq for breast cancer (214). For a complete coverage of available monoomic methodologies, see an excellent review by Casado-Pelaez et al. (215).

Despite these advances, challenges remain regarding the selectivity and efficacy of epigenetic therapies, especially for solid tumors. Combination therapies that integrate epigenetic drugs with immunotherapy or chemotherapy have been investigated to improve outcomes. Continued research is needed to better understand the specific epigenetic alterations across different cancers and to develop more effective and targeted therapies (19, 216).

Although advanced computational methods have greatly contributed to our understanding of the intricate network of epigenetic changes in cancer development, these analytical tools have limitations and challenges.

1. Need for large, high-quality datasets for training machine learning models.
2. The complexity of advanced AI models in biology, particularly deep learning approaches, often results in “black box” systems that are difficult to interpret, challenging researchers’ ability to extract meaningful biological insights and understand the underlying mechanisms of predictions.
3. Computational resource requirements for analyzing large-scale epigenomic datasets.
4. Challenges in integrating heterogeneous data types and handling missing data as well as epigenetic data can be contaminated with noise and artifacts, which can affect the accuracy of computational analyses.
5. Batch Effects: Differences in experimental conditions or sequencing platforms can introduce batch effects that can confound data analysis.
6. Benchmarking computational methods against real-world datasets is essential for assessing their performance and identifying areas of improvement. Another challenge is the lack of ground truth data for benchmarking.

## 5 Conclusions

Cancer epigenetics has emerged as a crucial field for understanding tumor development, progression, and potential therapeutic interventions. The reversible nature of epigenetic modifications makes them a promising target for cancer treatment. The key conclusions and future directions are as follows:

1. Epigenetic alterations, including DNA methylation, histone modifications, chromatin remodeling and noncoding RNAs, play fundamental roles in cancer initiation and progression.
2. Advanced technologies such as single-cell epigenomics and CRISPR-based epigenetic editing have revolutionized our understanding of tumor epigenetic landscapes and offer new avenues for research and therapy.
3. Epigenetic drugs (epi-drugs) have shown potential in clinical trials, particularly for hematological cancers; however, their efficacy in solid tumors remains a challenge.
4. Combination therapies involving epigenetic drugs with other treatment modalities (e.g., immunotherapy and chemotherapy) show promise in overcoming drug resistance and enhancing antitumor effects.
5. The development of more specific and potent epigenetic inhibitors with fewer side effects is a key area for future research.
6. Personalized epigenetic therapies based on individual patient epigenomic profiles represent a promising direction for precision medicine in cancer treatment.
7. Further research is needed to understand the complex interplay between genetic and epigenetic alterations in cancer, as well as the role of the tumor microenvironment in epigenetic regulation.
8. Ethical considerations surrounding epigenetic therapies, particularly regarding their potential heritable effects, need to be carefully addressed.

As the field of cancer epigenetics continues to evolve, interdisciplinary collaboration and technological innovations will be crucial for translating epigenetic insights into effective clinical strategies for cancer diagnosis, prognosis, and treatment.
